# Targeted Degradation of Class 1 HDACs With PROTACs is Highly Effective at Inducing DLBCL Cell Death

**DOI:** 10.1002/jha2.70127

**Published:** 2025-08-12

**Authors:** Abdullah Alraddadi, Joshua P. Smalley, Wael Alzahrani, Anes Saleh, Fares Al‐Mansour, Buwei He, Thong H. Cao, Sandrine Jayne, Martin Dyer, James T. Hodgkinson, Donald J. L. Jones, Shaun M. Cowley, Salvador Macip

**Affiliations:** ^1^ Mechanisms of Cancer and Aging Laboratory University of Leicester Leicester UK; ^2^ Department of Molecular and Cell Biology University of Leicester Leicester UK; ^3^ The Ernest and Helen Scott Haematological Research Institute Leicester Cancer Research Centre University of Leicester Leicester UK; ^4^ Leicester Institute of Structural and Chemical Biology School of Chemistry University of Leicester Leicester UK; ^5^ Leicester Van Geest MultiOMICS Facility Hodgkin Building University of Leicester Leicester UK; ^6^ Department of Cardiovascular Sciences College of Life Sciences University of Leicester Leicester UK; ^7^ Epi4health Faculty of Health Sciences Universitat Oberta de Catalunya Barcelona Spain; ^8^ BarcelonaBeta Brain Research Center Pasqual Maragall Foundation Barcelona Spain

**Keywords:** apoptosis, DLBCL, HDAC, HDAC inhibitors, PROTAC

## Abstract

Despite the good options for the management of Diffuse large B‐cell lymphoma (DLBCL), a significant percentage of patients either do not respond to current treatments or relapse after a short time. Thus, a wider palette of targeted therapeutic strategies is needed. Histone deacetylases (HDACs) inhibitors have shown promising responses in B‐cell malignancies, but their off‐target effects limit their efficiency. Here, we investigated the use of novel targeted therapeutics against class I HDACs to specifically induce cell death in DLBCL cells. We show that a proteolysis targeting chimera (PROTAC) that combined HDAC inhibitor CI‐994 and an IAP ligand had a strong effect in killing different DLBCL cell lines, being more effective in doing so than CI‐994 on its own. Moreover, we show that this was concomitant with the induction of DNA damage and apoptosis. A proteomics screen showed that the mechanism of induction of cell death by this PROTAC likely depends on the simultaneous activation of pro‐apoptotic proteins (such as PARP‐1, PDCD6IP, DAPk1, TP53BP1, and CACYBP) and the inhibition of pro‐survival pathways. We conclude that eliminating class I HDACs with specific PROTACs could be an effective and precise strategy for treating DLBCL that should be further tested for their potential clinical relevance.

**Trial Registration**: The authors have confirmed clinical trial registration is not needed for this submission.

## 1 Introduction

Diffuse large B‐cell lymphoma (DLBCL) is the most common type of non‐Hodgkin lymphoma in adults [1]. It accounts for 30%–40% of all lymphoid cancers and has a high level of heterogeneity in terms of clinical outcomes and genetic changes [2]. Although it can be observed at any age, DLCBL has an increased incidence in elderly people, with a median age of onset of 65, and is more common in men than in women [3, 4]. Currently, patients with DLBCL are treated by immunochemotherapy using the R‐CHOP regimen (cyclophosphamide, vincristine, doxorubicin, prednisone, and humanized monoclonal antibody directed at B‐cell marker CD20, rituximab), which has a curative rate of 60% [5]. However, around 15% of patients do not respond to R‐CHOP, and 25% will relapse after 2 years of initial therapy [6, 7]. These cases will be subjected to a second line of therapy, which includes a high dose of chemotherapy followed by autologous stem cell transplantation [8]. It remains a major challenge to identify resistance mechanisms to traditional treatments and to develop novel therapeutic options for this disease.

Epigenetic modifications are a mechanism to control gene expression without altering the DNA [9]. Among these, histone acetylation, which is regulated by histone acetyl transferases (HATs) and histone deacetylases (HDACs) [10], has been shown to be involved in many cellular processes, and its deregulation may contribute to diseases such as cancer [11, 12, 13]. HDACs are a family of transcriptional regulatory enzymes that deacetylate lysine residues on histone and nonhistone proteins to regulate gene expression [14]. The human genome contains 18 distinct HDACs, which are classified into two groups (zinc‐dependent or nicotinamide adenine dinucleotide [NAD]‐dependent) and five classes (I, IIa, IIb, III, and IV) [15].

Class I zinc‐dependent enzymes HDAC 1, 2, and 3 account for 50% of all cellular deacetylase activity and are required for B‐cell development [16]. In the pre‐B cell differentiation stage, HDAC1 and HDAC2 stimulate development, their inhibition resulting in cell cycle arrest and apoptosis [17]. Also, HDAC3 knockout mice had defective B cell maturation as well as impairments in VDJ recombination [18]. Moreover, alterations in HDAC expression have been linked to hematological cancers [19]. For instance, HDACs play a crucial role in several signaling pathways that enhance the survival of malignant cells [20].

Consistent with this, HDAC inhibitors (HDACi) have been shown to be clinically effective drugs in certain malignancies [21]. To date, five HDACi have received Food and Drug Administration (FDA) approval for cancer treatment, and further compounds are now being tested [22]. HDAC1/2 inhibitors trigger apoptosis in acute lymphoblastic leukemia, while HDAC3 inhibitors are more effective in T‐cell lymphoma cell lines, suggesting that selectively targeting HDAC1/2/3 might be beneficial in certain disorders [23]. Of note, nearly 30% of DLBCL patients have mutations in genes involved in the acetylation process, such as *CREBBP* and *EP300* [24, 25]. Thus, targeting the HDACs has been proposed to be a potential therapy for DLBCL.

New approaches that target HDAC1/2 and 3 utilizing proteolysis targeting chimeras (PROTACs) can degrade these enzymes instead of just inhibiting their activity [26]. PROTACs are heterobifunctional bispecific molecules consisting of an E3 ligase ligand, an inhibitor that binds to the target, and a linker area that connects these two capabilities, which can polyubiquitinate and selectively mark for proteasomal degradation any given protein [27]. We generated a series of PROTACs based on CI‐994, a class I HDAC inhibitor with high selectivity against HDACs 1–3 [28], using the E3 ligase VHL (JPS016) or the IAP ligand (JPS026), and they were shown to induce apoptosis in cancer cells [23, 26, 29]. In view of this, we studied the possibility of using these PROTACs on DLBCL cells. Our results show that they may enhance cell death when compared to the original HDACi, which may be important for the development of more effective HDAC therapies for DLBCL.

## 2 Materials and Methods

### 2.1 Cell Culture, Reagents, and PROTACs

OCI‐LY19 was cultured in flasks in IMDM with L‐glutamine (Gibco 12440053) supplemented with 20% FBS, 1% P/S (Sigma‐Aldrich M6250). RIVA and U2932 were maintained in culture using Roswell Park Memorial Institute 1640 Medium (RPMI) (Gibco, Life Technologies Ltd) supplemented with 10% v/v fetal bovine serum (FBS, Gibco; Life Technologies Ltd) and 1% v/v penicillin/streptomycin (P/S, Gibco; Life Technologies Ltd). All cell lines were incubated at 37°C in a humidified atmosphere with 5% carbon dioxide.

### 2.2 Cell Viability and Death Assessment

DLBCL cell lines were harvested when confluency was close to 80%. The cell pellet was resuspended in the proper medium and seeded into a 96‐well plate at 4 × 10^4^ cells per 100 µL. Cells were treated with the compounds or with DMSO for the control. Following the incubation, 10 µL of CellTiterGlo (CTG) reagent (Promega G7572) was added to each well and gently shaken. After that, the plate was incubated for 5 min at room temperature and was left in the dark. It was then that the plate was uploaded to the HidexSense plate reader, and the luminescent signal was measured. All data were normalized to the untreated sample, and the experiments were performed in duplicates and repeated at least two independent times. For cell death analysis, cells were treated with the drugs and were stained with Annexin‐V staining (Sigma‐Aldrich), and then fluorescence was detected using flow cytometry (Beckman Coulter Cytoflex) according to the manufacturer's protocol. Data was analyzed by FlowJo and presented using GraphPad Prism 9.0.

### 2.3 Western Blotting

Cells were seeded and treated for 24 h with corresponding drugs in a 6‐well plate at a density of 2.5 × 10^6^ per 5 mL. Cells were pelleted and washed twice with cold 1xPBS before being lysed. Then the cell pellets were resuspended in 100 µL of RIPA lysis buffer. Protease and Phosphatase inhibitors (Sigma‐Aldrich) were added to the lysis buffer in a 1:100 ratio and incubated for 20 min on ice. Then, the cell suspension was ruptured by syringing 3 times. Pellets were then mixed with an equivalent amount of 0.4 N H_2_SO_4_ for histone extraction and left overnight at 4°C; the protein content of the samples was determined using the Bradford assay (Thermo Fisher). Following that, Polyacrylamide gels were prepared and run using standard protocols. After the transfer, membranes were scanned using the Odyssey Imager (LI‐COR). Protein was measured using Image Studio software (LI‐COR) to quantify the densitometry of bands and normalized to the loading control. The antibodies that were used: α‐tubulin (Sigma, t5168), HDAC1(Abcam, 109411), HDAC2 (Merck Millipore, 05–814), HDAC3(Abcam, 32369), H3(Merck Millipore, 05–499), H3K56Ac (Active Motif, 39082), PARP (Cell Signaling Technology, 9542), γH2AX (Millipore, 05–636), XIAP (BD Biosciences, 610762), c‐IAP1 (Cell Signaling Technology, 7065S), cIAP2 (Abcam, ab23423).

### 2.4 Preparation of Samples for Mass Spectrometry

The cell pellet was resuspended in 600 µL of 1% ammonium deoxycholate (ADC) (Sigma‐Aldrich) and placed in a Homogenizer (Beadblaster 24 microtube homogenizer). After homogenization, the samples were centrifuged at 16,000 *g* for 20 min, and the supernatants were transferred into new labeled Eppendorf tubes. The sample's lysate was either used for a protein concentration experiment or stored at −80°C until required. To determine the concentration of protein, a bicinchoninic acid assay was used. BSA was used as a standard protein for quantifying the amount of protein in the samples. The BCA working solution was prepared by mixing 1.35 mL of solution A (0.8 g of sodium carbonate monohydrate and 0.16 mg of tartaric acid into 10 mL of water, pH = 11.25 with NaOH), 1.25 mL of B solution (1 g of bicinchoninic acid in 25 mL of water), and 50 µL of C solution (40 mg of copper sulphate in 1 mL of water). The top wells (A1 and A2) were loaded with 120 µL of water and 80 µL of BSA as a standard. Then, 100 µL of water was added to the wells from B1 to H1 and from B2 to H2. A serial dilution was made by transferring 100 µL from the top wells (standard), except the bottom wells (H1 and H2) were left the same. To load the samples, each well was loaded with 98 µL of water and 2 µL of sample in triplicate, followed by adding 100 µL of the working solution. After the plate was incubated for an hour at 65°C (Thermo Multiskan Ascent), the results were read at 562 nm by a spectrophotometer. Following the estimation of protein concentration using the BCA assay, equivalent amounts of protein (1000 µg) were transferred to Microcon Centrifugal Filter units (Milipore), followed by adding Ammonium bicarbonate (ABC) to dilute 1000 µg of protein into a final volume of 1000 µL. Then, the samples were incubated for 1 h at 65°C after adding Dithiothreitol (Sigma‐Aldrich) (15.4 mg of dithiothreitol into 100 µL of 50 mmol ammonium bicarbonate) at a final concentration of 20 mM/L. After that, Iodoacetamide (Sigma‐Aldrich) (18.5 mg of iodoacetamide into 200 µL of 50 mmol ammonium bicarbonate) at a concentration of 40 mM/L, followed by incubation for 1 h in the dark at room temperature. Next, 1 µg/µL Trypsin (20 µL of 50 mmol ammonium bicarbonate into a vial of 20 µg of trypsin [Roche]) was added to samples at a 1:25 ratio to protein content, 1 µg/µL, and left overnight at 37°C. The next day, the activity of trypsin was stopped by adding 0.1% formic acid (FA) to the samples. Empore Solid Phase Extraction Cartridges (3 M) were used for peptide extraction. After being labeled and washed with ethanol, the columns were emptied by gravity. Then, the columns were washed four times with 0.1% FA. After that, the samples were placed and flowed through the columns by gravity, followed by washing with 0.1% FA four times. To collect the samples, new labeled Eppendorf tubes were prepared and 600 µL of 60% Acetonitrile (Sigma‐Aldrich) was applied to the columns, followed by adding 600 µL of 80% acetonitrile (Sigma‐Aldrich) for elution by gravity. Following that, samples were centrifuged for 90 min using a speed vacuum centrifuge (Thermo, RC1010). Following snap freezing in liquid nitrogen, samples were placed in a freeze dryer (LyoDry Compact Benchtop, MechaTech) and left overnight. The following day, samples were reconstituted with 30 µL of 0.1% FA and stored at −80°C until required. After that, an o‐Phthaladehyde (OPA) assay was applied to identify the concentration of peptide in the samples. Firstly, in the standard wells A1 and A2 of the plate, 90 µL of water and 10 µL of 1 mg/mL ROCK peptide (20 mg of OPA [Fisher Scientific] were added to 250 µL of Dimethylformamide [Sigma‐Aldrich]). Secondly, 50 µL of water was added into wells B1 to H1 and B2 to H2. Next, the standard wells were serially diluted in 50 µL amounts, excluding the last wells, H1 and H2. In the sample wells, 49 µL of water and 1 µL of each sample were then added in triplicate. Then, to all of the wells, 100 µL of a boric acid mixture including 200 µL of ROCK peptide, 20 mL of boric acid, and 40 µL of mercaptoethanol was added, followed by a 5‐min incubation at room temperature before reading at an excitation of 340 nm and emission of 490 nm. Lastly, 5 µL of each sample was transferred to labeled 32 mm glass screw neck vials and diluted to be 1 µg/µL using 0.1% FA and alcohol dehydrogenase.

## 3 Results

### 3.1 PROTAC‐Based Degradation of Class I HDACs in DLBCL Cells

**FIGURE 1:**
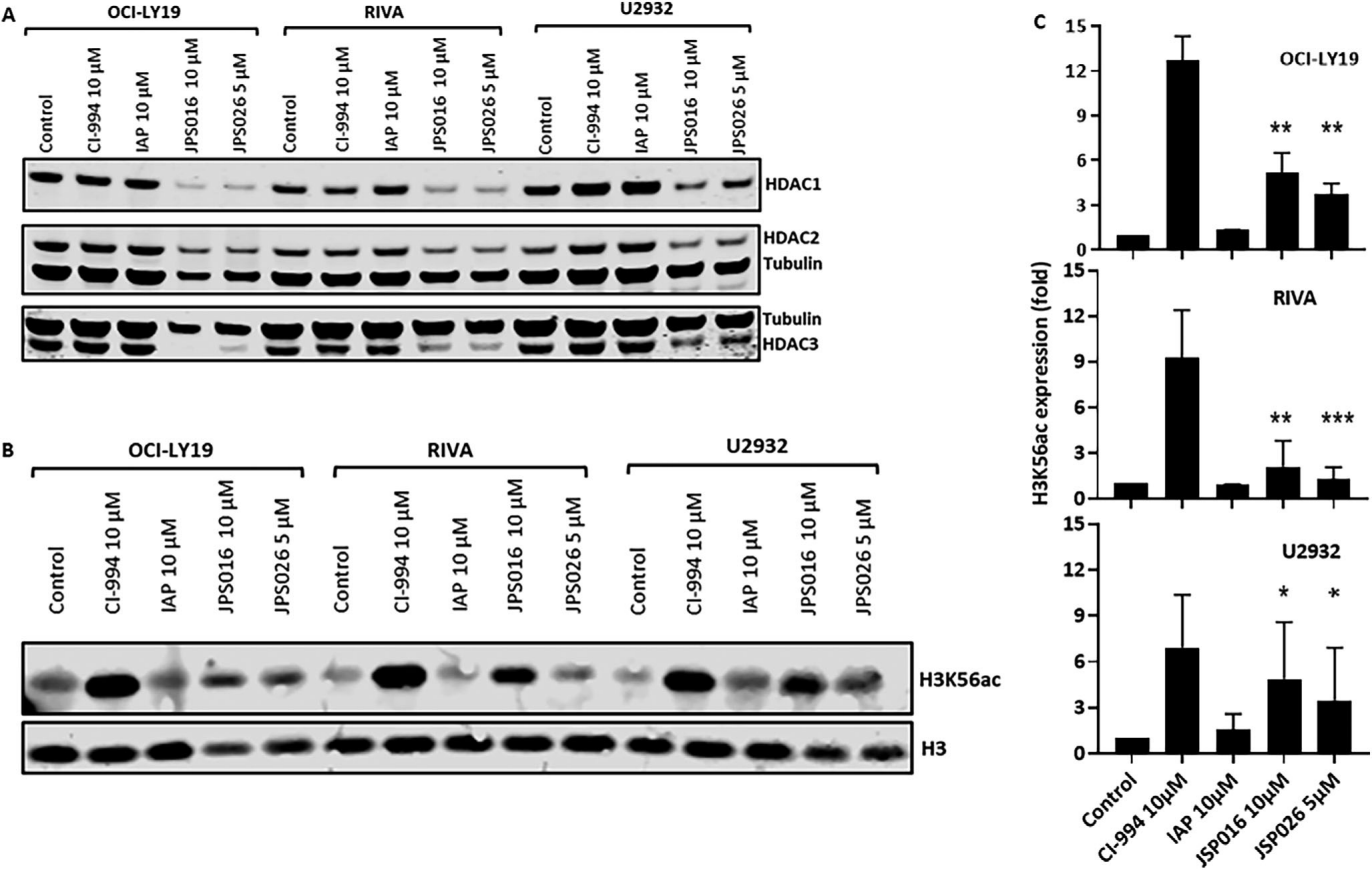
JPS016 and JPS026 PROTACs degrade class I HDACs in DLBCL. **(A)** Representative Western blots showing HDACs 1–3 protein expression in three DLBCL cell lines exposed to 10 µM of CI‐994, JPS016, IAP ligand, or 5 µM of JPS026 for 24 h. Control cells with treated with equal volumes of DMSO. Tubulin was used as loading control. (**B)** Representative Western blots showing expression H3K56Ac in the same cells. H3 was used as loading control. **(C)** Quantification of H3K56Ac levels, normalized to H3, in three independent experiments. Mean fold changes in expression (compared to controls) and standard deviations are plotted.

To investigate whether PROTACs against Class I HDACs could be a potential treatment for B cell malignancies, the DLBCL cell lines OCI‐LY19, RIVA, and U2932 were treated with the PROTACs JPS016 or JPS026 [23, 26, 29]. These cells were chosen as representative of different types of DLBCL: OCI‐LY19 comes from a germinal center DLBCL; RIVA (also known as RI‐1) is an ABC‐like lymphoma subtype, like U2932 (however, the latter has a mutated p53 while the former expresses the wild type form, which influences their sensitivity and response to chemotherapeutic drugs). Chemical HDACi CI‐994 was used as a positive control. As shown in Figure 1A, OCI‐LY19 and RIVA experienced a significant degradation of HDACs 1–3 after 24 h treatment with either JPS016 or JPS026, compared to a slight degradation in U2932. Neither CI‐994 nor the negative control, the IAP ligand alone, showed degradation of any of the HDACs in the three cell lines tested.

To confirm the PROTAC‐based degradation of HDACs in DLBCL, we measured the levels of Histone H3 Lysine 56 Acetylation (H3K56ac), a marker of newly synthesized chromatin and DNA repair, as well as Class I HDAC activity [30]. Both JPS016 and JPS026 increased H3K56ac levels in all cell lines tested, although at levels below those of CI‐994, while the IAP ligand did not change acetylation levels (Figure 1B,C). These results together confirm that JPS026 and JPS016 are effective at degrading class I HDACs and thus inhibit their activity in different DLBCL cell lines.

### 3.2 PROTACs Against Class I HDACs Reduce DLBCL Proliferation

**FIGURE 2:**
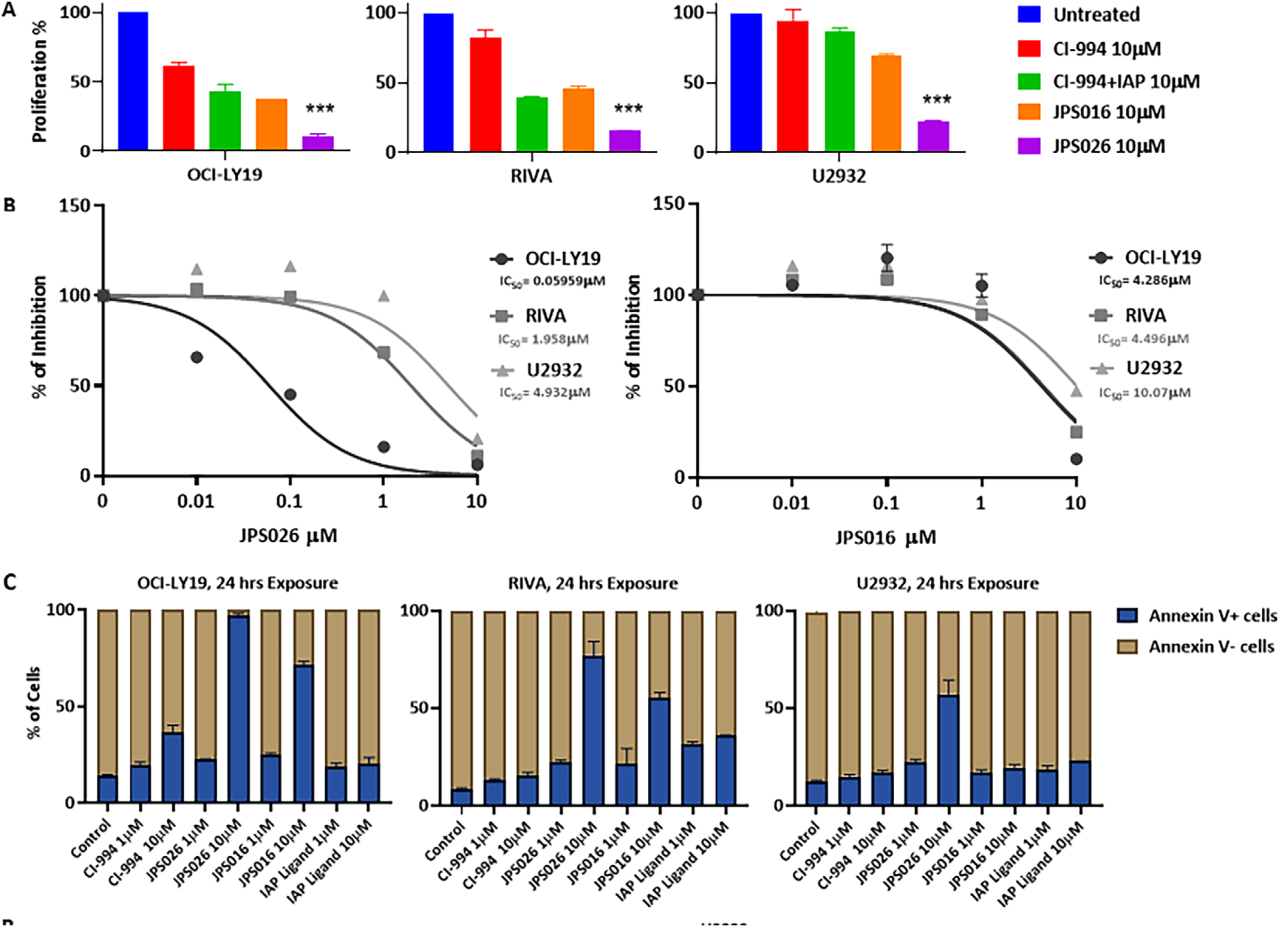
Effect of the PROTACs on the proliferation of DLBCL cell lines. (A) Cell viability in the DLBCL cells described in Figure 1, as measured by the CellTiterGlo assay. Mean values, normalized to the control treated with DMSO, ± standard deviation are plotted. *n* = 3. (B) Dose‐response curves of the same experiments. The percentage of inhibition and the IC50 were calculated using GraphPad Prism software. Data are expressed as mean ± standard deviation. (C) FACS analysis of Annexin V‐stained DLBCL cells treated with different concentrations of CI‐994, JPS026, JPS016 and IAP ligand for 24 h. Data were normalized to the control treated with DMSO and plotted as mean ± standard deviation. Statistical significance at was determined using 2‐way ANOVA with control sample: *** *p* < 0.001.

We next investigated the effects of the PROTAC‐based HDAC degradation on the proliferative potential of DLBCL cells. To this end, OCI‐LY19, RIVA, and U2932 were treated with JPS016 or JPS026 for 24 h. As shown in Figure 2A, JPS026 induced a reduction in cell viability, as measured by a CTG assay, of nearly 90% in OCI‐LY19, 80% in RIVA, and 70% in U2932. For JPS016, the reduction was less pronounced (60%, 50%, and 40%, respectively). These effects were observed at 10 µM, but not at lower concentrations (Figure S1). For comparison, 10 µM CI‐994 resulted in a 40% and 20% decrease in cell growth of OCI‐LY19 and RIVA, respectively, and no reduction in U2932, and the effect was only slightly increased by the addition of the IAP ligand. Also, our results show that JPS026 is more effective than JPS016, as confirmed by the dose‐response curves (Figure 2B), which showed IC_50_ of 0.05959 µM (OCI‐LY19), 1.958 µM (RIVA), and 4.932 µM (U2932) for JPS026, as opposed to 4.286, 4.496 µM, and 10.07 µM, respectively, for JPS016.

To better understand the nature of the inhibitory effect of JPS026 and JPS016 on DLBCL cell proliferation, we measured the induction of apoptosis using an Annexin‐V staining. Consistent with the results shown above, 10 µM of either PROTAC was able to induce apoptosis in all cell lines at percentages higher than those achieved with CI‐994, with U2932 being the most resistant, and JPS026 was more potent than JPS016 (Figure 2C). This shows that the PROTACs have a potent effect in reducing DLBCL proliferation and inducing apoptosis, above that of a widely used chemical inhibitor of HDACs.

### 3.3 JPS026 Downregulates IAPs in DLBCL

**FIGURE 3:**
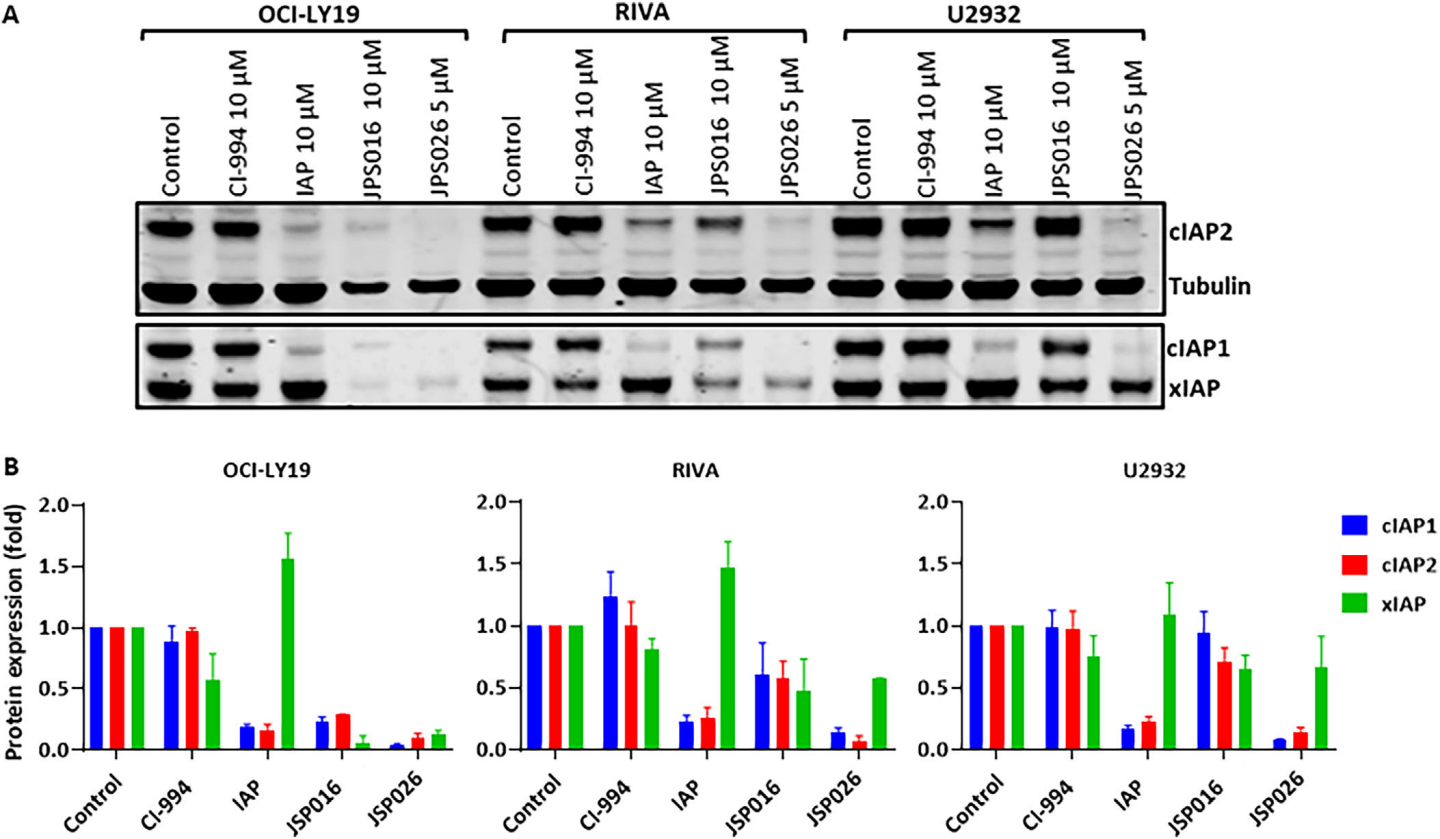
PROTACs induce degradation of IAPs. (A) Representative Western blots showing expression cIAP1, cIAP2 and xIAP in DLBCL cells exposed to 10 µM of CI‐994, JPS016, IAP ligand, and 5 µM of JPS026 for 24 h. Tubulin was used as loading control. (B). Quantification of the protein expression of the same blots (*n* = 3), normalized to the controls. Statistical significance at was determined using 2‐way ANOVA as compared to control sample. ns: not‐significant *p* > 0.05, * *p* < 0.05, ** *p* < 0.01, *** *p* < 0.001, ****p < 0.0001).

Our results show a stronger effect of JPS026 than JPS016 in inducing DLBCL cell death. Since the main difference between these two PROTACs is the E3 ligase used, we hypothesized that the IAP ligand present in JPS026 could itself be contributing to the cytotoxic effect, despite the fact that the IAP ligand alone showed no effects when used as a control in the experiments described above. The ligand could be auto‐ubiquitinated by the action of the PROTACs themselves, which could result in loss of the E3 proteins of the family [29] and contribute to cell death [31, 32]. To investigate this possibility, we measured the protein levels of c‐IAP1, c‐IAP2, and x‐IAP in DLBCL cells after PROTAC treatment. As expected, CI‐994 had no relevant effect on the IAPs in any cell line (Figure 3). When they were treated with the IAP ligand alone, cIAP1 and cIAP2 were reduced in all cells, while the levels of xIAP remained unchanged, consistent with the ligand inducing auto‐ubiquitination of some of these proteins. JPS026 was able to induce a reduction of cIAP1, cIAP2, and xIAP, an effect that was stronger in OCI‐LY19, suggesting that the IAP ligand being part of a PROTAC could contribute to the induction of apoptosis by blocking the IAP proteins. This could explain the higher cytotoxicity of JPS026. Of note, JPS026 seemed to have a greater effect on IAP inhibition than the IAP ligand alone, even at lower concentrations. Interestingly, a similar effect was also seen in some cells, to a certain extent, with JPS016, which suggests that the effect could be independent of the IAP component and in part related to the mechanism of action of the PROTAC.

### 3.4 Potential Mechanisms of Action of JPS026 in DLBCL

**FIGURE 4:**
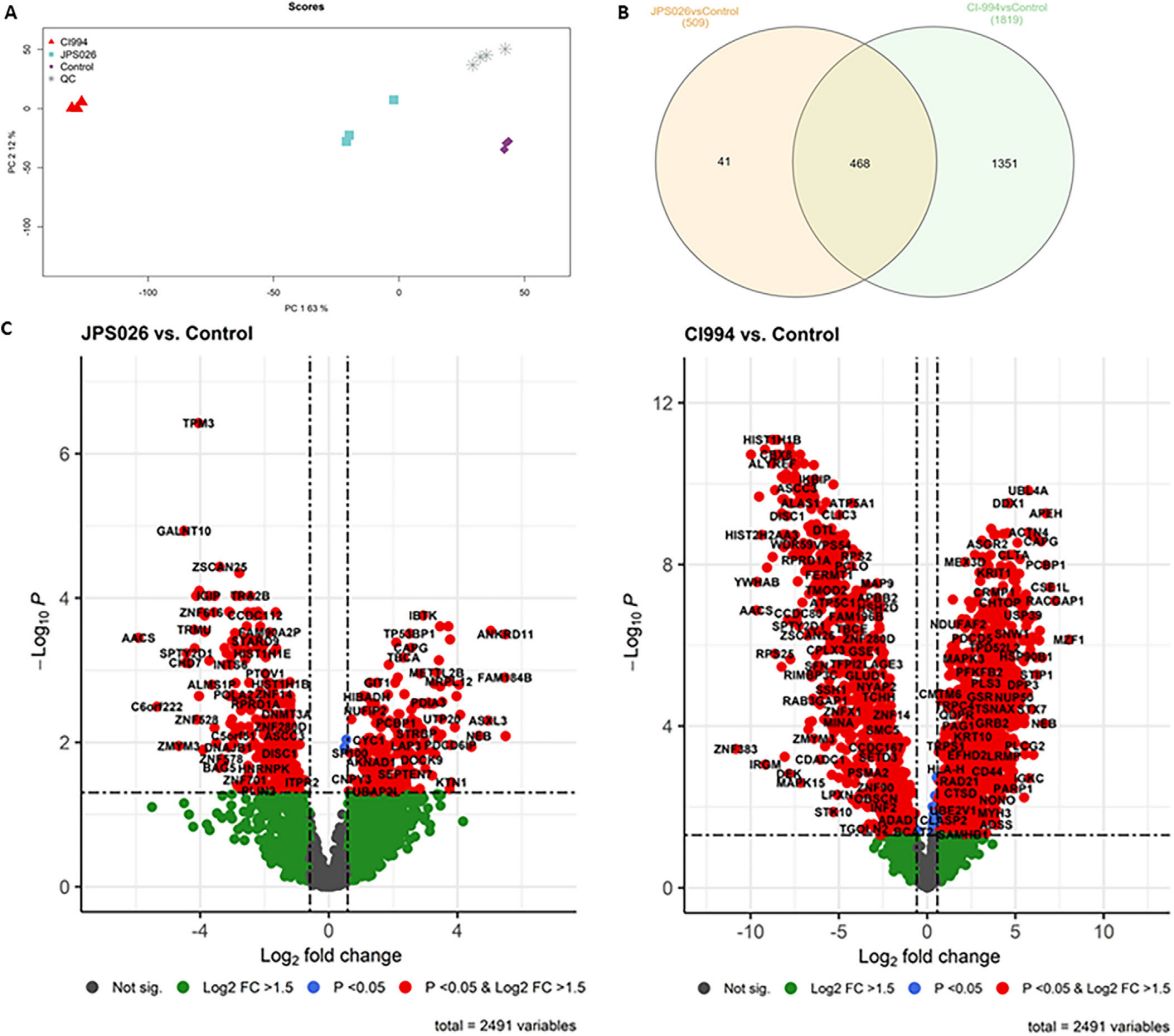
Mass spectrometry analysis of the effects of JPS026 on OCI‐LY19. (A) Principal Component Analysis of OCI‐LY19 treated with 10 µM of CI‐994 or 5 µM of JPS026 for 24 h, compared to control samples. Samples are colored to indicate the differences between the groups. *N* = 3 per control and treated groups, and *N* = 4 for quality control (QC). (B) Venn Diagram of the upregulated and downregulated proteins identified in JPS026 and CI‐994, with these common in the middle. (C) Volcano plots showing the distribution of all proteins expressed in OCI‐LY19 treated with CI‐994 (left) or JPS026 (right), compared to DMSO‐treated controls.

Finally, we performed a mass spectrometry screen in order to better understand the strong pro‐apoptotic effects that JPS026 exerts on DLBCL. To this end, we chose the OCI‐LY19 cells, which showed the highest sensitivity in our experiments, and treated them with either CI‐994 or JPS026. Principal component analysis showed a clustering of the replicates for each treatment, which grouped close to each other, confirming the quality of the samples (Figure 4A). A total of 1819 (1131 upregulated and 688 downregulated) and 509 (207 upregulated and 302 downregulated) proteins were identified as significantly changed by CI‐994 or JPS026, respectively, of which 468 were shared between the two treatments, and 41 only changed by JPS026, suggesting that they are specifically related to the effects of the PROTAC (Figure 4B,C; Table S1). Furthermore, pathway analysis through the Reactome platform showed that proteins exclusively regulated by JPS026 were involved in pathways related to cell cycle, programmed cell death, and chromatin organization (Figure S2). Further pathway analysis using Panther showed upregulated pathways related to the p53 pathway and thus potentially involved in apoptosis (Figure S3). Indeed, several of the proteins upregulated by JPS026 but not CI‐994 have been associated with cellular apoptotic processes, such as PARP‐1, PDCD6IP, DAPK1, TP53BP1, and CACYBP [33, 34, 35, 36, 37, 38, 39, 40, 41, 42, 43]. On the other hand, 14‐3‐3β/α, a potential therapeutic target in different cancers [44, 45], was downregulated. The 14‐3‐3 proteins are important regulators of the p53 pathway and thus can act as tipping cellular responses towards apoptosis [46]. Similarly, pro‐survival proteins, such as MYCBP, a transcription factor overexpressed in various human malignancies [47, 48, 49, 50, 51], were downregulated. Downregulation of MYCBP has been shown to inhibit cancer cell migration, invasion, and metastasis [52, 53, 54].

These data provide a mechanistic explanation for the strong apoptotic response of JPS026, which is likely based on the simultaneous activation of pro‐apoptotic anti‐survival signals. This is consistent with the fact that DLBCL cells can be particularly sensitive to the inhibition of pro‐survival signals [55]. All the results together show the complexity of the effects of a class I HDAC‐specific PROTAC on DLBCL cells, which are likely to depend both on the inhibition of HDAC expression and activity, and other unrelated functions.

## 4 Discussion

PROTACs have received a lot of attention as a novel approach for targeting oncogenic proteins with unprecedented efficacy [56, 57, 58]. They exhibit several advantages over traditional small‐molecule inhibitors (SMIs). First, by acting catalytically to trigger degradation of proteins, PROTACs could require lower doses, thereby reducing systemic drug exposure and potential side effects [59]. Second, PROTACs can be designed to target proteins deemed to be “undruggable”, and they have the potential to have a longer duration of action [60]. Finally, PROTACs can overcome resistance to drugs or compensatory increases caused by mutations in the protein of interest, which often happens in SMI treatments [61].

Several PROTACs have been developed to target proteins that are involved in the development and progression of malignant hematologic tumors [62, 63, 64, 65, 66, 67, 68, 69]. Some have been demonstrated to be particularly effective in killing cancer cells and suppressing tumor growth [70]. Given the fact that class I HDAC inhibition has been shown to be toxic in DLBCL cells, we proposed that a PROTAC targeting class I HDACs could have a strong clinical potential, and we took advantage of previously generated tools to test this hypothesis. HDACs are a particularly interesting target in B cell malignancies, since their inhibition has been shown to abrogate pro‐survival pathways and thus induce cell death with high efficacy [71].

JPS026 and JPS016, which use the HDACi CI‐994 to direct binding to their targets, have been shown to be heterobifunctional degraders of Class I HDACs [26, 72], and our results confirm that this is the case in DLBCL cells as well, with JPS026 being more effective than JPS016. Of note, both PROTACs were less able than CI‐994 to inhibit HDAC activity, as measured by reduction in histone acetylation, but, nevertheless, were shown to be more toxic to the cells. This suggests that the absence of the HDACs is more detrimental for DLBCL cells than the simple inhibition of their function, a fact that should be further explored in future studies.

The toxicity exerted by the PROTACs on DLBCL cells was found to be dependent on the induction of apoptosis, which could be in part triggered by an increase in DNA damage. Previous studies also showed induction of cell death by JPS026 and JPS016 in HCT116 colon cancer cells [29]. CI‐994 has previously been shown to have wide anti‐tumoral efficacy and induce apoptosis [73], which is consistent with our findings. However, the stronger effects of the PROTACs suggest that the compounds exert their effects through mechanisms complementary to the suppression of HDAC activity in cells. In this line, we observed that DLBCL cells were considerably more sensitive to JPS026 than JPS016, which is compatible with the fact that the E3 ligase chosen for the design of the PROTAC may itself play a role in the induction of cell death, as previously suggested [74]. Of note, we used high concentrations of the PROTACs in our experiments, based on our previous results, to be able to fully appreciate their cellular effects. In order to fully understand the mechanisms of cell death triggered in our models, future studies should focus on a more detailed exploration of potential off‐target effects at these doses, exploring binding affinities with other proteins. We saw no effect of JPS026 on other families of HDACs (data not shown), but we cannot rule out that other unrelated proteins could also be affected and thus contribute to the toxic effect on DLBCL cells.

Indeed, our experiments showed that JPS026, which uses IAP ligand to activate different E3 ligases, downregulated several of these IAP ligases in a stronger manner than JPS016, in which the E3 ligase is VHL. Inactivating IAPs can result in apoptosis induction [75], and IAP proteins are often overexpressed in cancer and have a role in the survival of tumor cells, resistance to chemotherapy, and disease progression [76]. This could help explain the different potency of the two compounds, as simultaneous degradation of both IAP and HDAC may have a stronger effect. This is consistent with the fact that IAP‐based PROTACs are considered to have a stronger clinical potential than other compounds [77]. In our previous studies, the IAP ligand, either on its own or as part of JPS026, reduced the levels of c‐IAP2, which results in a potent apoptosis induction in HCT116 cells, suggesting that the decrease in levels of c‐IAP2 seen may be caused by autoubiquitination and contributes to the induction of cell death [29]. Moreover, since the IAP ligand used in JPS026 is non‐specific, it can bind various members of the IAP family that have E3 ligase activity, and this may play a role in increasing its potency [78].

Other mechanistic explanations of the particular toxicity of JPS026 in DLBCL could be inferred from the list of proteins that the treatment upregulates and downregulates exclusively when compared to the chemical inhibitors. Apart from the exclusive induction of the pro‐apoptotic proteins discussed above, which could explain the enhanced toxicity, several histones were upregulated in JPS026‐treated cells. Interestingly, Histone H2B type 1‐L (HIST1H2BL), which has been associated with apoptotic chromatin, resulting in the regulation of chromatin structure during apoptosis [79], was found exclusively upregulated by JPS026 and not CI‐994. Also strongly induced was the Inhibitor of Bruton tyrosine kinase protein (IBTK), which blocks BTK, a non‐receptor kinase that plays an important role in carcinogenic signaling and is required for the growth and survival of cancerous B cells [80]. Of note, the total number of proteins modulated by JPS026 is less than that affected by CI‐994, which might be indicative of a higher selectivity.

Our results strengthen the case for HDAC inhibition as a potential treatment for DLBCL and support previous experiments showing a superior effect of PROTACs over SMIs. The clinical potential of these PROTACs should be tested next. Of note, the different DLBCL cells we utilized had a positive but mixed response to the PROTACs, which suggests that cancer cell heterogeneity may play a role in defining the sensitivity to HDAC elimination. Indeed, OCI‐LY19, derived from germinal center B‐cell‐like (GCB) DLBCL, displays distinct genetic alterations (BCL2, EZH2, CREBBP), unique epigenetic changes, and activation of BCR signaling and PI3K/AKT/mTOR pathways [81, 82]. On the other hand, U2932 and RIVA originate from a more aggressive activated B‐cell‐like (ABC) DLBCL. They have mutations in MYD88, CD79B, and CARD11, and they also have different epigenetic features and activation of the NF‐κB pathway [83, 84]. Identifying the factors that define this response may provide stratification markers for the potential clinical use of these compounds.

## Author Contributions

Experiments were designed and analysed by SM and AA, with help from SC and MJD. JTH designed the PROTACs and JPS synthesized them. Experiments were performed by AA, with help from WA, BH, AS and FA. Mass spectrometry experiments were designed, performed and analysed by SM, AA, THC and DJLJ. The manuscript was written by SM and AA and reviewed by all authors.

## Conflicts of Interest

JTH, JPS and SMC are named inventors in the following patents and patent applications that include JPS026 and JPS016: EP4093730B1, JPWO2021148811A5, AU2021211930A1, US20230120211A1, CA3165051A1 and WO2021148811A1.

## Supporting information




**Figure S1. Effect of the PROTACs on the proliferation of DLBCL Cell lines**. (A) Cell viability in the DLBCL cells treated with different concentrations of the PROTACS or the IAP as a negative control, as measured by the CellTiterGlo assay. Mean values, normalized to the control treated with DMSO, +/‐ standard deviation are plotted. N = 3.
**Figure S2**. Reactome analysis of the pathways in which the proteins that have significantly changed in OCI‐LY19 treated with JP026 are grouped.
**Figure S3**. PANTHER analysis of the pathways in which the proteins that have significantly changed in OCI‐LY19 treated with JP026 are grouped.
**Table S1**. List of the proteins that change after JP026 or CI‐994 treatment in OCI‐LY19 cells. In bold, the main proteins changed by JP026 but not CI‐994.

## Data Availability

All data generated in this study is either included in the supplementary material or available upon request.
