# Correction to “Targeted Degradation of Class 1 HDACs With PROTACs is Highly Effective at Inducing DLBCL Cell Death”

**DOI:** 10.1002/jha2.70159

**Published:** 2025-10-21

A. Alraddadi, J. P. Smalley, W. Alzahrani, et al. “Targeted Degradation of Class 1 HDACs With PROTACs is Highly Effective at Inducing DLBCL Cell Death,” *EJHaem* 6, no. 4 (2025): e70127, https://doi.org/10.1002/jha2.70127.

Co‐author Martin J. S. Dyer was incorrectly listed as Martin Dyer.

We apologize for this error.

