# Supplementary material for: Targeted Degradation of Class 1 HDACs With PROTACs is Highly Effective at Inducing DLBCL Cell Death
Source: EJHaem. 2025 Aug 12;6(4):e70127. doi: 10.1002/jha2.70127 (PMC12342057; doi:10.1002/jha2.70127)
Supplement: Supplementary file 1 — Figure S1. Effect of the PROTACs on the proliferation of DLBCL Cell lines. (A) Cell viability in the DLBCL cells treated with different concentrations of the PROTACS or the IAP as a negative control, as measured by the CellTiterGlo assay. Mean values, normalized to the control treated with DMSO, +/‐ standard deviation are plotted. N = 3. Figure S2. Reactome analysis of the pathways in which the proteins that have significantly changed in OCI‐LY19 treated with JP026 are grouped. Figure S3. PANTHER analysis of the pathways in which the proteins that have significantly changed in OCI‐LY19 treated with JP026 are grouped. Table S1. List of the proteins that change after JP026 or CI‐994 treatment in OCI‐LY19 cells. In bold, the main proteins changed by JP026 but not CI‐994. [file JHA2-6-e70127-s001.docx]

**
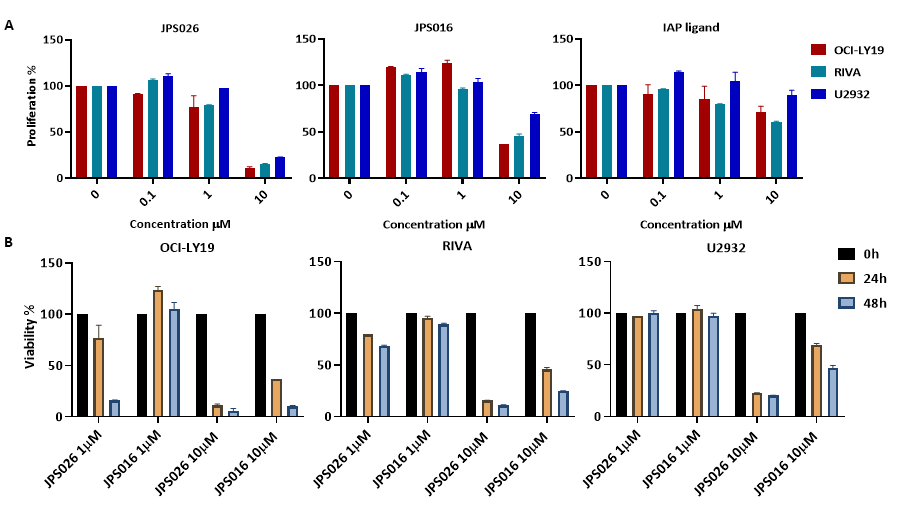
**

**Supplementary Figure 1. Effect of the PROTACs on the proliferation of DLBCL Cell lines.** (A) Cell viability in the DLBCL cells treated with different concentrations of the PROTACS or the IAP as a negative control, as measured by the CellTiterGlo assay. Mean values, normalized to the control treated with DMSO, +/- standard deviation are plotted. N=3.

**
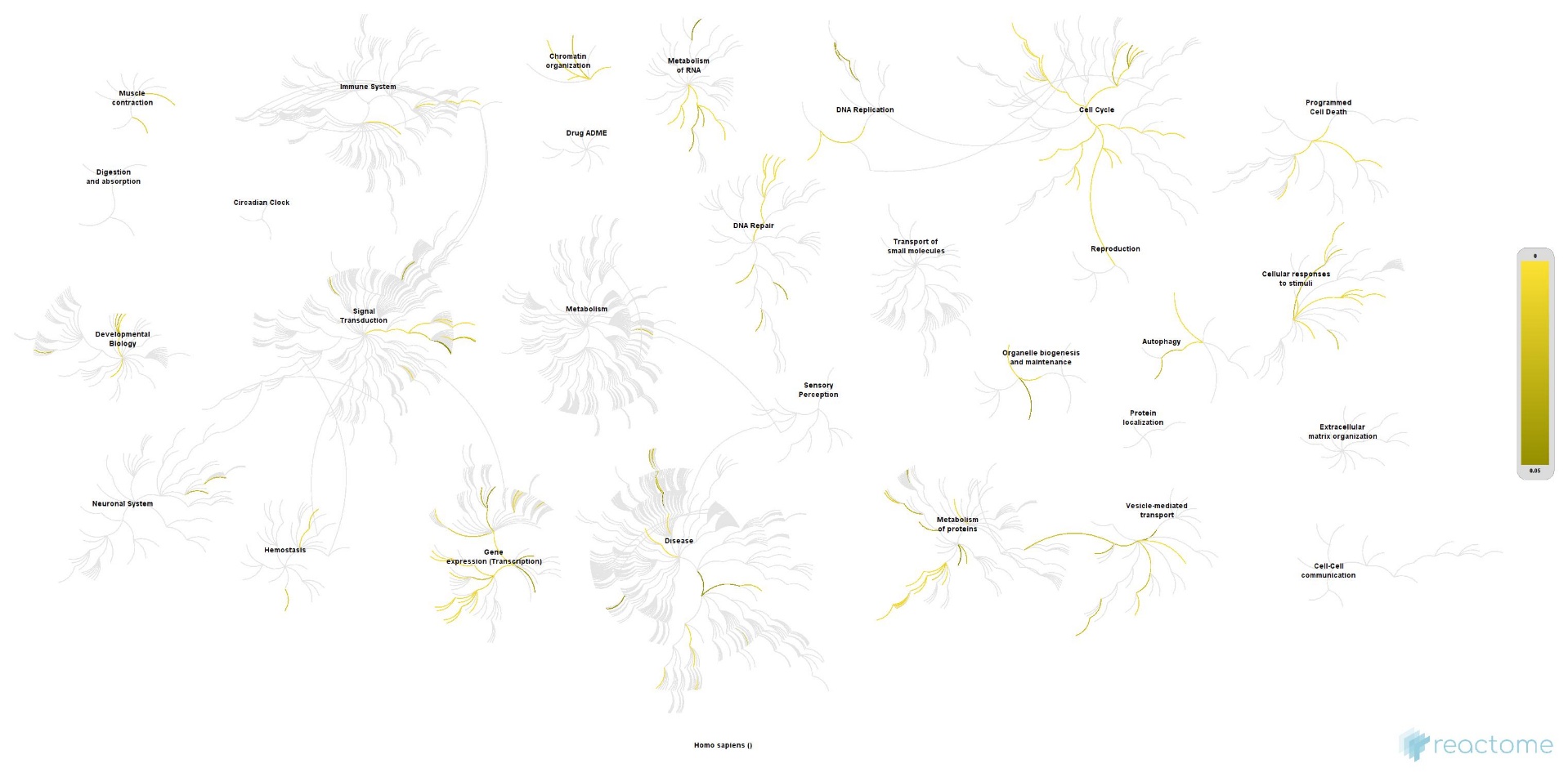
**

**Supplementary Figure 2.** Reactome analysis of the pathways in which the proteins that have significantly changed in OCI-LY19 treated with JP026 are grouped.

**
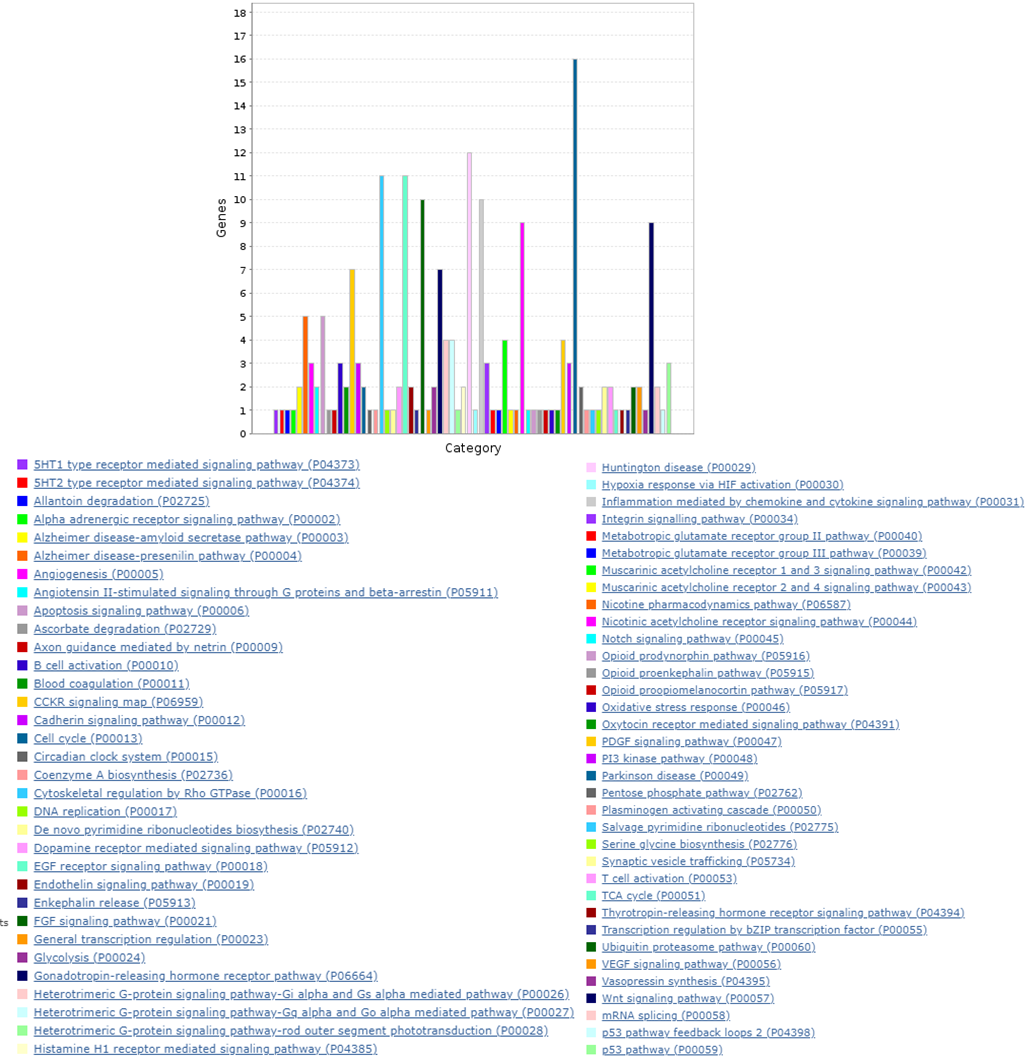
**

**Supplementary Figure 3.** PANTHER analysis of the pathways in which the proteins that have significantly changed in OCI-LY19 treated with JP026 are grouped.

**Supplementary Table 1.** List of the proteins that change after JP026 or CI-994 treatment in OCI-LY19 cells. In bold, the main proteins changed by JP026 but not CI-994.

| **Gene name** | **Protein name** | **P.value** | **Fold change** | **Change** |
| --- | --- | --- | --- | --- |
| **ANKRD11** | **Ankyrin repeat domain-containing protein 11** | **0.0003164** | **45.28167991** | **Up** |
| **PARP1** | **Poly [ADP-ribose] polymerase 1** | **0.0081731** | **45.13368133** | **Up** |
| FAM184B | Protein FAM184B | 0.0012588 | 44.54711948 | Up |
| MAPT | Microtubule-associated protein tau | 0.000284 | 32.74348285 | Up |
| **ASXL3** | **Putative Polycomb group protein ASXL3** | **0.0049783** | **30.95818367** | **Up** |
| NEB | Nebulin | 0.0081258 | 25.41288853 | Up |
| PAPLN | Papilin | 0.0115365 | 21.64484883 | Up |
| EPB41 | Protein 4.1 | 0.0041489 | 16.94229107 | Up |
| **EIF4H** | **Eukaryotic translation initiation factor 4H** | **0.0015855** | **16.48588752** | **Up** |
| UCP2 | Mitochondrial uncoupling protein 2 | 0.0022835 | 15.65005676 | Up |
| LCP1 | Plastin-2 | 0.0061742 | 15.14354349 | Up |
| KTN1 | Kinectin | 0.0358895 | 14.0665808 | Up |
| **PDCD6IP** | **Programmed cell death 6-interacting protein** | **0.0108661** | **13.73250467** | **Up** |
| **INO80** | **DNA helicase INO80** | **0.0003738** | **13.62378046** | **Up** |
| RTN1 | Reticulon-1 | 0.0444932 | 13.51886723 | Up |
| MRPL12 | 39S ribosomal protein L12_ mitochondrial | 0.0014372 | 13.32597856 | Up |
| HNRNPL | Heterogeneous nuclear ribonucleoprotein L | 0.0002465 | 13.15595115 | Up |
| **CACYBP** | **Calcyclin-binding protein** | **0.0027416** | **11.49047555** | **Up** |
| UTP20 | Small subunit processome component 20 homolog | 0.0047549 | 11.46897812 | Up |
| EWSR1 | RNA-binding protein EWS | 0.0077812 | 11.28210928 | Up |
| C7orf60 | Probable methyltransferase BTM2 homolog | 0.0016704 | 11.05504463 | Up |
| TJP2 | Tight junction protein ZO-2 | 0.0002465 | 10.984779 | Up |
| NCKAP5L | Nck-associated protein 5-like | 0.0007278 | 10.70361515 | Up |
| MZF1 | Myeloid zinc finger 1 | 0.0088048 | 10.55234674 | Up |
| METTL2B | Methyltransferase-like protein 2B | 0.0010816 | 10.21998807 | Up |
| **CASP7** | **Caspase-7** | **0.0146566** | **9.89344891** | **Up** |
| RTN4 | Reticulon-4 | 0.0069557 | 9.789773359 | Up |
| CTR9 | RNA polymerase-associated protein CTR9 homolog | 0.0015479 | 9.75725331 | Up |
| ATP5O | ATP synthase subunit O_ mitochondrial | 0.002912 | 9.487296988 | Up |
| ZAP70 | Tyrosine-protein kinase ZAP-70 | 0.0134384 | 9.315494629 | Up |
| ATXN2L | Ataxin-2-like protein | 0.0287784 | 9.262321835 | Up |
| TRDN | Triadin | 0.002912 | 8.833958301 | Up |
| PDIA3 | Protein disulfide-isomerase A3 | 0.0028068 | 8.563973545 | Up |
| WTAP | Pre-mRNA-splicing regulator WTAP | 0.023176 | 8.004981718 | Up |
| DNAH2 | Dynein heavy chain 2_ axonemal | 0.002912 | 7.895692317 | Up |
| TBCB | Tubulin-folding cofactor B | 0.0128275 | 7.870572193 | Up |
| **HIST1H2BL** | **Histone H2B type 1-L** | **0.0094752** | **7.860787276** | **Up** |
| **IBTK** | **Inhibitor of Bruton tyrosine kinase** | **0.000174** | **7.652299062** | **Up** |
| DOCK9 | Dedicator of cytokinesis protein 9 | 0.0175741 | 7.412967564 | Up |
| BAG2 | BAG family molecular chaperone regulator 2 | 0.0118044 | 7.394691322 | Up |
| KIF2C | Kinesin-like protein KIF2C | 0.0064131 | 7.390533344 | Up |
| TBCC | Tubulin-specific chaperone C | 0.0011138 | 7.147057154 | Up |
| PTBP1 | Polypyrimidine tract-binding protein 1 | 0.0441649 | 7.112536861 | Up |
| C1orf173 | Uncharacterized protein C1orf173 | 0.0149999 | 6.954952152 | Up |
| KIF16B | Kinesin-like protein KIF16B | 0.0053014 | 6.753439346 | Up |
| ADAMTS19 | A disintegrin and metalloproteinase with thrombospondin motifs 19 | 0.002912 | 6.74801651 | Up |
| STRBP | Spermatid perinuclear RNA-binding protein | 0.0075918 | 6.651857643 | Up |
| CCRN4L | Nocturnin | 0.0271447 | 6.450346222 | Up |
| NGLY1 | Peptide-N(4)-(N-acetyl-beta-glucosaminyl)asparagine amidase | 0.0029102 | 6.357654314 | Up |
| KIF20A | Kinesin-like protein KIF20A | 0.0070289 | 6.313733937 | Up |
| MYH10 | Myosin-10 | 0.0096289 | 6.268464089 | Up |
| ELP4 | Isoform 2 of Elongator complex protein 4 | 0.0481917 | 6.163160466 | Up |
| EDC4 | Enhancer of mRNA-decapping protein 4 | 0.0047802 | 5.996051267 | Up |
| HAUS4 | HAUS augmin-like complex subunit 4 | 0.0263808 | 5.992670159 | Up |
| EIF4A3 | Eukaryotic initiation factor 4A-III | 0.0035054 | 5.928238148 | Up |
| CAPG | Macrophage-capping protein | 0.0004904 | 5.871746964 | Up |
| CHMP4B | Charged multivesicular body protein 4b | 0.0096289 | 5.797075624 | Up |
| SNX30 | Sorting nexin-30 | 0.0138862 | 5.775676187 | Up |
| RAB39A | Ras-related protein Rab-39A | 0.0098747 | 5.684151197 | Up |
| **TP53BP1** | **Tumor suppressor p53-binding protein 1** | **0.0003106** | **5.677263573** | **Up** |
| ADAD1 | Adenosine deaminase domain-containing protein 1 | 0.0077949 | 5.599362009 | Up |
| RBM39 | RNA-binding protein 39 | 0.0138529 | 5.576785005 | Up |
| RPS27L | 40S ribosomal protein S27-like | 0.022509 | 5.576624396 | Up |
| IRF2BP2 | Interferon regulatory factor 2-binding protein 2 | 0.0055681 | 5.494576666 | Up |
| KIAA1217 | Sickle tail protein homolog | 0.008377 | 5.384069277 | Up |
| IGLL5 | Immunoglobulin lambda-like polypeptide 5 | 0.020839 | 5.361463073 | Up |
| FILIP1L | Filamin A-interacting protein 1-like | 0.039016 | 5.330327895 | Up |
| GTF2I | General transcription factor II-I | 0.0463039 | 5.320935233 | Up |
| ATG16L1 | Autophagy-related protein 16-1 | 0.0234251 | 5.310981933 | Up |
| LAP3 | Cytosol aminopeptidase | 0.0111086 | 5.28144863 | Up |
| TPP2 | Tripeptidyl-peptidase 2 | 0.0340152 | 5.265162384 | Up |
| SEPTEN7 | Septin-7 | 0.0275734 | 5.196092651 | Up |
| SOD1 | Superoxide dismutase [Cu-Zn] | 0.0162159 | 5.189396574 | Up |
| PBK | Lymphokine-activated killer T-cell-originated protein kinase | 0.0117841 | 5.171947915 | Up |
| STIP1 | Stress-induced-phosphoprotein 1 | 0.0401448 | 5.166002333 | Up |
| DYNC1LI2 | Cytoplasmic dynein 1 light intermediate chain 2 | 0.0307463 | 5.10039437 | Up |
| ALDOC | Fructose-bisphosphate aldolase C | 0.002148 | 5.08970737 | Up |
| TBCA | Tubulin-specific chaperone A | 0.0006646 | 4.993905538 | Up |
| CHKB | Choline/ethanolamine kinase | 0.0359559 | 4.905598628 | Up |
| LDHB | L-lactate dehydrogenase B chain | 0.0391315 | 4.897848086 | Up |
| THSD7A | Thrombospondin type-1 domain-containing protein 7A | 0.0127913 | 4.888388394 | Up |
| RNF40 | E3 ubiquitin-protein ligase BRE1B | 0.0098747 | 4.88517353 | Up |
| SRRM2 | Serine/arginine repetitive matrix protein 2 | 0.0384501 | 4.794200783 | Up |
| NDUFB11 | NADH dehydrogenase [ubiquinone] 1 beta subcomplex subunit 11_ mitochondrial | 0.0099532 | 4.783441574 | Up |
| PLCB4 | 1-phosphatidylinositol 4_5-bisphosphate phosphodiesterase beta-4 | 0.0126961 | 4.779830709 | Up |
| EIF4ENIF1 | Eukaryotic translation initiation factor 4E transporter | 0.0444303 | 4.703807426 | Up |
| NUP50 | Nuclear pore complex protein Nup50 | 0.0348171 | 4.672163875 | Up |
| MDH1 | Malate dehydrogenase_ cytoplasmic | 0.0437737 | 4.637794594 | Up |
| ARPC1B | Actin-related protein 2/3 complex subunit 1B | 0.0040652 | 4.581650006 | Up |
| COPG1 | Coatomer subunit gamma-1 | 0.0247554 | 4.579582243 | Up |
| CEP57 | Centrosomal protein of 57 kDa | 0.0040452 | 4.480101649 | Up |
| ZNF443 | Zinc finger protein 443 | 0.0076681 | 4.446213346 | Up |
| TIMM44 | Mitochondrial import inner membrane translocase subunit TIM44 | 0.0376902 | 4.426945824 | Up |
| RNASET2 | Ribonuclease T2 | 0.0012596 | 4.422876584 | Up |
| BZW1 | Basic leucine zipper and W2 domain-containing protein 1 | 0.0296766 | 4.410555379 | Up |
| COL2A1 | Collagen alpha-1(II) chain | 0.0145052 | 4.346565686 | Up |
| PABPC1 | Polyadenylate-binding protein 1 | 0.0485219 | 4.343366102 | Up |
| PCBP1 | Poly(rC)-binding protein 1 | 0.0053846 | 4.278097468 | Up |
| ACO2 | Aconitate hydratase_ mitochondrial | 0.0004122 | 4.275557783 | Up |
| RAB1A | Ras-related protein Rab-1A | 0.0302652 | 4.253445986 | Up |
| CRKL | Crk-like protein | 0.0063475 | 4.189611943 | Up |
| CLINT1 | Clathrin interactor 1 | 0.0099532 | 4.186964207 | Up |
| NCOA7 | Nuclear receptor coactivator 7 | 0.0485219 | 4.181870813 | Up |
| PUS7 | Pseudouridylate synthase 7 homolog | 0.0014855 | 4.176967487 | Up |
| DDX47 | Probable ATP-dependent RNA helicase DDX47 | 0.0328081 | 4.024158079 | Up |
| FAP | Seprase | 0.0056952 | 4.01051444 | Up |
| PRDX6 | Peroxiredoxin-6 | 0.0329246 | 4.003412079 | Up |
| NASP | Nuclear autoantigenic sperm protein | 0.0361066 | 3.956223042 | Up |
| RPS12 | 40S ribosomal protein S12 | 0.0330162 | 3.944206231 | Up |
| PPP4R2 | Serine/threonine-protein phosphatase 4 regulatory subunit 2 | 0.0485812 | 3.915598003 | Up |
| KIF20B | Kinesin-like protein KIF20B | 0.0473981 | 3.832121108 | Up |
| SGTA | Small glutamine-rich tetratricopeptide repeat-containing protein alpha | 0.0394812 | 3.812255108 | Up |
| TUFM | Elongation factor Tu_ mitochondrial | 0.0169109 | 3.715619069 | Up |
| CHP2 | Calcineurin B homologous protein 2 | 0.0049805 | 3.708139825 | Up |
| CDC42 | Cell division control protein 42 homolog | 0.0228117 | 3.68137704 | Up |
| NAV2 | Neuron navigator 2 | 0.0110218 | 3.641636157 | Up |
| AHSG | Alpha-2-HS-glycoprotein | 0.0008362 | 3.639466026 | Up |
| RPA1 | Replication protein A 70 kDa DNA-binding subunit | 0.002179 | 3.57032869 | Up |
| FAM98B | Isoform 2 of Protein FAM98B | 0.0169109 | 3.517032153 | Up |
| **CYCS** | **Cytochrome c** | **0.0127913** | **3.482268259** | **Up** |
| THRA | Thyroid hormone receptor alpha | 0.0359716 | 3.430653988 | Up |
| CEP78 | Centrosomal protein of 78 kDa | 0.0452794 | 3.409769909 | Up |
| AP1S1 | AP-1 complex subunit sigma-1A | 0.0341102 | 3.351295888 | Up |
| F11 | Isoform 2 of Coagulation factor XI | 0.0106887 | 3.309254526 | Up |
| MPHOSPH8 | M-phase phosphoprotein 8 | 0.0138862 | 3.28474626 | Up |
| SF3B2 | Splicing factor 3B subunit 2 | 0.0035054 | 3.265826066 | Up |
| ETFA | Electron transfer flavoprotein subunit alpha_ mitochondrial | 0.0055581 | 3.240905617 | Up |
| CHAMP1 | Chromosome alignment-maintaining phosphoprotein 1 | 0.0271447 | 3.228829421 | Up |
| NMD3 | 60S ribosomal export protein NMD3 | 0.0454049 | 3.22762922 | Up |
| ALKBH5 | RNA demethylase ALKBH5 | 0.0330162 | 3.166245633 | Up |
| TNKS1BP1 | 182 kDa tankyrase-1-binding protein | 0.0190378 | 3.163776496 | Up |
| ATL3 | Atlastin-3 | 0.0138396 | 3.089022249 | Up |
| MAP2 | Microtubule-associated protein 2 | 0.0265034 | 3.075061167 | Up |
| **DIDO1** | **Death-inducer obliterator 1** | **0.0041469** | **3.061962324** | **Up** |
| KNTC1 | Kinetochore-associated protein 1 | 0.0123674 | 3.060195353 | Up |
| UBXN1 | UBX domain-containing protein 1 | 0.0061742 | 3.055085428 | Up |
| ZFC3H1 | Zinc finger C3H1 domain-containing protein | 0.0086812 | 3.048825197 | Up |
| IARS2 | Isoleucine--tRNA ligase_ mitochondrial | 0.0186282 | 3.004481546 | Up |
| **HIST1H2AB** | **Histone H2A type 1-B/E** | **0.0185344** | **2.994553687** | **Up** |
| RPS6KA2 | Ribosomal protein S6 kinase alpha-2 | 0.0054678 | 2.992921231 | Up |
| ARPP19 | cAMP-regulated phosphoprotein 19 | 0.022509 | 2.948435934 | Up |
| DPY19L1 | Probable C-mannosyltransferase DPY19L1 | 0.0361066 | 2.928411883 | Up |
| SMARCE1 | SWI/SNF-related matrix-associated actin-dependent regulator of chromatin subfamily E member 1 | 0.0099142 | 2.889724678 | Up |
| GAPDH | Glyceraldehyde-3-phosphate dehydrogenase | 0.0446258 | 2.885752099 | Up |
| ITGB2 | Integrin beta-2 | 0.0491111 | 2.85908735 | Up |
| POLR2B | DNA-directed RNA polymerase II subunit RPB2 | 0.0446258 | 2.849247939 | Up |
| GIT1 | ARF GTPase-activating protein GIT1 | 0.0014855 | 2.830310501 | Up |
| CCDC64B | Bicaudal D-related protein 2 | 0.0138464 | 2.800227323 | Up |
| UBAP2L | Ubiquitin-associated protein 2-like | 0.0482174 | 2.799957093 | Up |
| VBP1 | Prefoldin subunit 3 | 0.0478683 | 2.792071503 | Up |
| C7orf63 | Uncharacterized protein C7orf63 | 0.0053846 | 2.75893583 | Up |
| UFM1 | Ubiquitin-fold modifier 1 | 0.0042237 | 2.736633085 | Up |
| SMAD2 | Mothers against decapentaplegic homolog 2 | 0.0193538 | 2.736001834 | Up |
| NDRG3 | Protein NDRG3 | 0.0234251 | 2.723412117 | Up |
| ARGLU1 | Arginine and glutamate-rich protein 1 | 0.002912 | 2.718633745 | Up |
| AIMP1 | Isoform 2 of Aminoacyl tRNA synthase complex-interacting multifunctional protein 1 | 0.0336452 | 2.673782934 | Up |
| HNRNPH1 | Heterogeneous nuclear ribonucleoprotein H | 0.0127046 | 2.626714706 | Up |
| NANP | N-acylneuraminate-9-phosphatase | 0.0040652 | 2.622126694 | Up |
| ESYT3 | Extended synaptotagmin-3 | 0.0296072 | 2.611267033 | Up |
| SLC25A42 | Mitochondrial coenzyme A transporter SLC25A42 | 0.0492582 | 2.597641288 | Up |
| FBL | rRNA 2'-O-methyltransferase fibrillarin | 0.0395003 | 2.575189216 | Up |
| TXNRD1 | Thioredoxin reductase 1_ cytoplasmic | 0.0216804 | 2.565439536 | Up |
| SMC5 | Structural maintenance of chromosomes protein 5 | 0.0382778 | 2.565221733 | Up |
| GSTO1 | Glutathione S-transferase omega-1 | 0.0367358 | 2.53362013 | Up |
| GNAI3 | Guanine nucleotide-binding protein G(k) subunit alpha | 0.0199504 | 2.513931111 | Up |
| IDH3B | Isocitrate dehydrogenase [NAD] subunit beta_ mitochondrial | 0.0384501 | 2.511373572 | Up |
| TMC6 | Transmembrane channel-like protein 6 | 0.0371128 | 2.505563836 | Up |
| **PDCD5** | **Programmed cell death protein 5** | **0.0028719** | **2.496072113** | **Up** |
| AKNAD1 | Protein AKNAD1 | 0.018794 | 2.487307624 | Up |
| CDC42BPB | Serine/threonine-protein kinase MRCK beta | 0.0399948 | 2.482019086 | Up |
| PCM1 | Pericentriolar material 1 protein | 0.0099532 | 2.470343731 | Up |
| CCDC50 | Isoform 2 of Coiled-coil domain-containing protein 50 | 0.029726 | 2.443705819 | Up |
| CTNNA1 | Catenin alpha-1 | 0.038957 | 2.443419432 | Up |
| GRWD1 | Glutamate-rich WD repeat-containing protein 1 | 0.0086454 | 2.438247358 | Up |
| RABEP1 | Rab GTPase-binding effector protein 1 | 0.0014855 | 2.437799484 | Up |
| IL12RB2 | Interleukin-12 receptor subunit beta-2 | 0.0323156 | 2.433391581 | Up |
| CEBPB | CCAAT/enhancer-binding protein beta | 0.034492 | 2.375832278 | Up |
| **CYC1** | **Cytochrome c1_ heme protein_ mitochondrial** | **0.0093879** | **2.369555653** | **Up** |
| CAPZB | F-actin-capping protein subunit beta | 0.0293963 | 2.349703898 | Up |
| ICAM3 | Intercellular adhesion molecule 3 | 0.0086942 | 2.344519942 | Up |
| RBM42 | RNA-binding protein 42 | 0.0138464 | 2.340313617 | Up |
| NIT2 | Omega-amidase NIT2 | 0.0327583 | 2.332041096 | Up |
| GSE1 | Genetic suppressor element 1 | 0.0375233 | 2.314732007 | Up |
| HIBADH | 3-hydroxyisobutyrate dehydrogenase_ mitochondrial | 0.0023452 | 2.264075809 | Up |
| MMRN1 | Multimerin-1 | 0.0463039 | 2.240557989 | Up |
| CCDC144B | Coiled-coil domain-containing protein 144B | 0.0256513 | 2.207558385 | Up |
| NUFIP2 | Nuclear fragile X mental retardation-interacting protein 2 | 0.0036537 | 2.189584401 | Up |
| GLRX2 | Glutaredoxin-2_ mitochondrial | 0.0478683 | 2.174545305 | Up |
| SF3B5 | Splicing factor 3B subunit 5 | 0.0127913 | 2.153988386 | Up |
| PNPT1 | Polyribonucleotide nucleotidyltransferase 1_ mitochondrial | 0.0160895 | 2.108873618 | Up |
| LARS | Leucine--tRNA ligase_ cytoplasmic | 0.0230245 | 2.100098635 | Up |
| MYOF | Myoferlin | 0.0022728 | 2.092029763 | Up |
| LNP | Protein lunapark | 0.0102739 | 2.089618572 | Up |
| NARS | Asparagine--tRNA ligase_ cytoplasmic | 0.0463039 | 2.041955429 | Up |
| RSPH10B2 | Radial spoke head 10 homolog B2 | 0.0185086 | 2.038180504 | Up |
| ESAM | Endothelial cell-selective adhesion molecule | 0.0441511 | 2.012473355 | Up |
| MRPL40 | 39S ribosomal protein L40_ mitochondrial | 0.0375845 | 1.968104812 | Up |
| CD70 | CD70 antigen | 0.0478167 | 1.942115737 | Up |
| RAB3C | Ras-related protein Rab-3C | 0.0401448 | 1.868746522 | Up |
| CHORDC1 | Cysteine and histidine-rich domain-containing protein 1 | 0.0138862 | 1.848718259 | Up |
| CCDC88A | Girdin | 0.0342304 | 1.800617229 | Up |
| CNPY3 | Protein canopy homolog 3 | 0.0322777 | 1.634336979 | Up |
| CCDC132 | Coiled-coil domain-containing protein 132 | 0.0047656 | 1.626053964 | Up |
| SP100 | Nuclear autoantigen Sp-100 | 0.0141002 | 1.57933643 | Up |
| EIF2S2 | Eukaryotic translation initiation factor 2 subunit 2 | 0.0473091 | 1.540738493 | Up |
| PPFIBP1 | Liprin-beta-1 | 0.0091781 | 1.458016818 | Up |
| H2AFV | Histone H2A.V | 0.0118165 | 1.402729344 | Up |
| HOMER1 | Homer protein homolog 1 | 0.0332831 | 0.595992307 | Down |
| ITPR2 | Inositol 1_4_5-trisphosphate receptor type 2 | 0.0358895 | 0.56296902 | Down |
| EMD | Emerin | 0.0472537 | 0.537340571 | Down |
| KCNMA1 | Calcium-activated potassium channel subunit alpha-1 | 0.0106887 | 0.532307472 | Down |
| HIST1H4A | Histone H4 | 0.0342304 | 0.530327909 | Down |
| HIST1H2BA | Histone H2B type 1-A | 0.0261693 | 0.524581102 | Down |
| ZSCAN32 | Zinc finger and SCAN domain-containing protein 32 | 0.029999 | 0.518468063 | Down |
| FAM9A | Protein FAM9A | 0.0330162 | 0.514441339 | Down |
| SHROOM2 | Protein Shroom2 | 0.032498 | 0.501139087 | Down |
| DTL | Denticleless protein homolog | 0.0368333 | 0.494602513 | Down |
| SARDH | Sarcosine dehydrogenase_ mitochondrial | 0.0435401 | 0.494576273 | Down |
| PROSC | Proline synthase co-transcribed bacterial homolog protein | 0.021093 | 0.491209867 | Down |
| UQCRC1 | Cytochrome b-c1 complex subunit 1_ mitochondrial | 0.0302652 | 0.473096717 | Down |
| ZNF573 | Zinc finger protein 573 | 0.0201693 | 0.471837133 | Down |
| TAF1 | Transcription initiation factor TFIID subunit 1 | 0.0272556 | 0.465421209 | Down |
| SMG6 | Telomerase-binding protein EST1A | 0.0401448 | 0.462563353 | Down |
| TAGLN2 | Transgelin-2 | 0.0149999 | 0.460723221 | Down |
| LAGE3 | L antigen family member 3 | 0.0131578 | 0.45910256 | Down |
| ZFP69B | Zinc finger protein ZFP69B | 0.0106887 | 0.457177139 | Down |
| FAT2 | Protocadherin Fat 2 | 0.0403196 | 0.456658168 | Down |
| APLP2 | Amyloid-like protein 2 | 0.0117794 | 0.455741021 | Down |
| CTSW | Cathepsin W | 0.0141002 | 0.453325264 | Down |
| C8orf47 | Uncharacterized protein C8orf47 | 0.0081968 | 0.451669682 | Down |
| BDP1 | Transcription factor TFIIIB component B'' homolog | 0.0320096 | 0.449660675 | Down |
| PRKCA | Protein kinase C alpha type | 0.0087327 | 0.448867828 | Down |
| MRPS25 | 28S ribosomal protein S25_ mitochondrial | 0.0086454 | 0.448524563 | Down |
| SAMD9 | Sterile alpha motif domain-containing protein 9 | 0.041556 | 0.44781709 | Down |
| MYH9 | Myosin-9 | 0.0076034 | 0.446404909 | Down |
| MS4A14 | Membrane-spanning 4-domains subfamily A member 14 | 0.0076681 | 0.446127181 | Down |
| PXT1 | Peroxisomal testis-specific protein 1 | 0.0076025 | 0.445832458 | Down |
| F8 | Coagulation factor VIII | 0.0075918 | 0.445754248 | Down |
| PPID | Peptidyl-prolyl cis-trans isomerase D | 0.0380527 | 0.445046257 | Down |
| CAT | Catalase | 0.0149538 | 0.444785678 | Down |
| DCN | Decorin | 0.0325829 | 0.443265537 | Down |
| MYL6 | Isoform Smooth muscle of Myosin light polypeptide 6 | 0.0162159 | 0.440802183 | Down |
| RBM47 | RNA-binding protein 47 | 0.0056952 | 0.44069779 | Down |
| AIPL1 | Aryl-hydrocarbon-interacting protein-like 1 | 0.0053846 | 0.439524651 | Down |
| RPL9 | 60S ribosomal protein L9 | 0.0026275 | 0.437772123 | Down |
| NOXIN | Nitric oxide-inducible gene protein | 0.0117663 | 0.437596676 | Down |
| PHACTR4 | Isoform 4 of Phosphatase and actin regulator 4 | 0.0041731 | 0.43659318 | Down |
| PNKD | Isoform 2 of Probable hydrolase PNKD | 0.0041489 | 0.436453738 | Down |
| ABI3BP | Target of Nesh-SH3 | 0.0035054 | 0.435101044 | Down |
| BEND5 | BEN domain-containing protein 5 | 0.0022728 | 0.435005435 | Down |
| GBX2 | Homeobox protein GBX-2 | 0.0033088 | 0.434736488 | Down |
| HSH2D | Hematopoietic SH2 domain-containing protein | 0.002973 | 0.434150161 | Down |
| RAB4A | Ras-related protein Rab-4A | 0.0023291 | 0.432842731 | Down |
| APBB2 | Amyloid beta A4 precursor protein-binding family B member 2 | 0.0022835 | 0.432744576 | Down |
| KIF13A | Kinesin-like protein KIF13A | 0.0396501 | 0.432081777 | Down |
| MAP9 | Microtubule-associated protein 9 | 0.0014553 | 0.431769436 | Down |
| ATP10D | Probable phospholipid-transporting ATPase VD | 0.0187378 | 0.431606611 | Down |
| SRSF1 | Serine/arginine-rich splicing factor 1 | 0.0254552 | 0.431270038 | Down |
| WDR66 | WD repeat-containing protein 66 | 0.0141827 | 0.427867623 | Down |
| PDE4DIP | Myomegalin | 0.005407 | 0.427270827 | Down |
| DNAH17 | Dynein heavy chain 17_ axonemal | 0.0055581 | 0.421337711 | Down |
| MYH15 | Myosin-15 | 0.0455664 | 0.417423973 | Down |
| DNHD1 | Dynein heavy chain domain-containing protein 1 | 0.0089855 | 0.415295304 | Down |
| ZNF445 | Zinc finger protein 445 | 0.0297454 | 0.414773741 | Down |
| HNRNPM | Heterogeneous nuclear ribonucleoprotein M | 0.0090608 | 0.4134119 | Down |
| EEF2 | Elongation factor 2 | 0.0061574 | 0.412684261 | Down |
| NPC2 | Epididymal secretory protein E1 | 0.0093764 | 0.412549493 | Down |
| HSPA8 | Heat shock cognate 71 kDa protein | 0.0180692 | 0.408647179 | Down |
| SLMAP | Sarcolemmal membrane-associated protein | 0.037258 | 0.405955976 | Down |
| ZNF546 | Zinc finger protein 546 | 0.0136811 | 0.404935047 | Down |
| ULK3 | Serine/threonine-protein kinase ULK3 | 0.0022908 | 0.402039576 | Down |
| DNMT3A | DNA (cytosine-5)-methyltransferase 3A | 0.0040652 | 0.401444537 | Down |
| HDGFRP3 | Hepatoma-derived growth factor-related protein 3 | 0.0420283 | 0.400748654 | Down |
| FIP1L1 | Pre-mRNA 3'-end-processing factor FIP1 | 0.0101048 | 0.398137609 | Down |
| NXF2 | Nuclear RNA export factor 2 | 0.0061574 | 0.388929796 | Down |
| OGDH | 2-oxoglutarate dehydrogenase_ mitochondrial | 0.0108661 | 0.388594726 | Down |
| ASCC3 | Activating signal cointegrator 1 complex subunit 3 | 0.0081258 | 0.385297363 | Down |
| ROR1 | Tyrosine-protein kinase transmembrane receptor ROR1 | 0.0138862 | 0.385148075 | Down |
| ASPH | Isoform 3 of Aspartyl/asparaginyl beta-hydroxylase | 0.0083186 | 0.385075011 | Down |
| ABCA13 | ATP-binding cassette sub-family A member 13 | 0.0076034 | 0.383875153 | Down |
| HSP90AB3P | Putative heat shock protein HSP 90-beta-3 | 0.0047768 | 0.383822996 | Down |
| DOCK6 | Dedicator of cytokinesis protein 6 | 0.0232222 | 0.378291085 | Down |
| CHN2 | Beta-chimaerin | 0.029726 | 0.377160704 | Down |
| GGA1 | ADP-ribosylation factor-binding protein GGA1 | 0.036715 | 0.377034339 | Down |
| PNN | Pinin | 0.0307463 | 0.375129207 | Down |
| PLIN3 | Perilipin-3 | 0.0101025 | 0.374901485 | Down |
| NCL | Nucleolin | 0.0045058 | 0.373198067 | Down |
| AXDND1 | Axonemal dynein light chain domain-containing protein 1 | 0.0015544 | 0.372315344 | Down |
| LIMCH1 | Isoform 3 of LIM and calponin homology domains-containing protein 1 | 0.0141827 | 0.37086271 | Down |
| HMGB3 | High mobility group protein B3 | 0.0216804 | 0.36907975 | Down |
| LMNB1 | Lamin-B1 | 0.0112479 | 0.367677371 | Down |
| ANKRD65 | Ankyrin repeat domain-containing protein 65 | 0.0234251 | 0.367150869 | Down |
| HNRNPU | Heterogeneous nuclear ribonucleoprotein U | 0.0071404 | 0.36673568 | Down |
| ZC3H13 | Zinc finger CCCH domain-containing protein 13 | 0.002912 | 0.366732126 | Down |
| H2AFX | Histone H2AX | 0.0026786 | 0.366347813 | Down |
| HSPA2 | Heat shock-related 70 kDa protein 2 | 0.0106887 | 0.365390084 | Down |
| VCP | Transitional endoplasmic reticulum ATPase | 0.002912 | 0.364669693 | Down |
| SYNE1 | Isoform 7 of Nesprin-1 | 0.0109616 | 0.364508006 | Down |
| DDX26B | Protein DDX26B | 0.0174376 | 0.36217728 | Down |
| SHMT2 | Serine hydroxymethyltransferase_ mitochondrial | 0.0125408 | 0.361654768 | Down |
| KCNQ2 | Potassium voltage-gated channel subfamily KQT member 2 | 0.0192828 | 0.361388304 | Down |
| IGFN1 | Isoform 5 of Immunoglobulin-like and fibronectin type III domain-containing protein 1 | 0.0242897 | 0.360469241 | Down |
| CEP135 | Centrosomal protein of 135 kDa | 0.0127581 | 0.360148879 | Down |
| ZNF280D | Zinc finger protein 280D | 0.0060669 | 0.355099069 | Down |
| VDAC2 | Voltage-dependent anion-selective channel protein 2 | 0.0062914 | 0.354805801 | Down |
| HIST1H1B | Histone H1.5 | 0.0015544 | 0.354029465 | Down |
| CSNK1D | Casein kinase I isoform delta | 0.029726 | 0.353455699 | Down |
| RBBP6 | E3 ubiquitin-protein ligase RBBP6 | 0.0049805 | 0.350673366 | Down |
| MAP7D2 | MAP7 domain-containing protein 2 | 0.0342304 | 0.349882266 | Down |
| GNAT3 | Guanine nucleotide-binding protein G(t) subunit alpha-3 | 0.029576 | 0.347672305 | Down |
| SCAF8 | Protein SCAF8 | 0.0075918 | 0.347222715 | Down |
| RBMX | RNA-binding motif protein_ X chromosome | 0.0136811 | 0.346881992 | Down |
| DISC1 | Disrupted in schizophrenia 1 protein | 0.0141904 | 0.346172449 | Down |
| CNBP | Isoform 4 of Cellular nucleic acid-binding protein | 0.002912 | 0.345283474 | Down |
| ZNF112 | Zinc finger protein 112 | 0.0041489 | 0.345215277 | Down |
| RPAP3 | RNA polymerase II-associated protein 3 | 0.0423401 | 0.339460108 | Down |
| FERMT1 | Fermitin family homolog 1 | 0.0120757 | 0.338987121 | Down |
| RPLP0P6 | 60S acidic ribosomal protein P0-like | 0.0062566 | 0.338890683 | Down |
| TAX1BP1 | Tax1-binding protein 1 | 0.0300867 | 0.337403297 | Down |
| RFC5 | Replication factor C subunit 5 | 0.0149999 | 0.336824392 | Down |
| ATOX1 | Copper transport protein ATOX1 | 0.0126582 | 0.331838195 | Down |
| ZNF726 | Zinc finger protein 726 | 0.0056952 | 0.331370181 | Down |
| ZNF732 | Zinc finger protein 732 | 0.0376399 | 0.331322752 | Down |
| PDZK1 | Na(+)/H(+) exchange regulatory cofactor NHE-RF3 | 0.0485219 | 0.330651739 | Down |
| TES | Testin | 0.0270284 | 0.330338824 | Down |
| MARCKSL1 | MARCKS-related protein | 0.0004988 | 0.321574059 | Down |
| PCLO | Protein piccolo | 0.0015888 | 0.320658918 | Down |
| IQGAP2 | Ras GTPase-activating-like protein IQGAP2 | 0.0132471 | 0.31777417 | Down |
| ATP5A1 | ATP synthase subunit alpha_ mitochondrial | 0.0001574 | 0.317528722 | Down |
| CAPRIN2 | Caprin-2 | 0.0041292 | 0.317167168 | Down |
| SNRPF | Small nuclear ribonucleoprotein F | 0.0101048 | 0.317136835 | Down |
| IVL | Involucrin | 0.0160546 | 0.316870759 | Down |
| ATP1A1 | Sodium/potassium-transporting ATPase subunit alpha-1 | 0.0254552 | 0.315635284 | Down |
| NUCB2 | Nucleobindin-2 | 0.0312867 | 0.315004434 | Down |
| MDH2 | Malate dehydrogenase_ mitochondrial | 0.0016105 | 0.311963201 | Down |
| **CDK11A** | **Cyclin-dependent kinase 11A** | **0.0005425** | **0.311670792** | **Down** |
| COX4I1 | Cytochrome c oxidase subunit 4 isoform 1_ mitochondrial | 0.0312021 | 0.311515619 | Down |
| OR52K1 | Olfactory receptor 52K1 | 0.0022835 | 0.310690192 | Down |
| SRSF4 | Serine/arginine-rich splicing factor 4 | 0.0026275 | 0.307101492 | Down |
| PDLIM1 | PDZ and LIM domain protein 1 | 0.0110218 | 0.306359077 | Down |
| ENO2 | Gamma-enolase | 0.0022835 | 0.306039858 | Down |
| ZNF14 | Zinc finger protein 14 | 0.002148 | 0.305826028 | Down |
| GRPEL1 | GrpE protein homolog 1_ mitochondrial | 0.0406141 | 0.301073903 | Down |
| PTGES3 | Prostaglandin E synthase 3 | 0.012227 | 0.297358766 | Down |
| MYO15A | Unconventional myosin-XV | 0.0376902 | 0.296872508 | Down |
| NFATC4 | Nuclear factor of activated T-cells_ cytoplasmic 4 | 0.0219664 | 0.295762351 | Down |
| SRP9 | Signal recognition particle 9 kDa protein | 0.0120757 | 0.295334656 | Down |
| KIAA0430 | Meiosis arrest female protein 1 | 0.0179854 | 0.293868876 | Down |
| CCBL2 | Kynurenine--oxoglutarate transaminase 3 | 0.0103333 | 0.292812107 | Down |
| PFN1 | Profilin-1 | 0.0087594 | 0.29171384 | Down |
| RGN | Regucalcin | 0.0085606 | 0.28459873 | Down |
| CYP1A1 | Cytochrome P450 1A1 | 0.0144035 | 0.284468537 | Down |
| CBLB | E3 ubiquitin-protein ligase CBL-B | 0.005579 | 0.284277983 | Down |
| ZNF254 | Zinc finger protein 254 | 0.005407 | 0.282987197 | Down |
| NEFL | Neurofilament light polypeptide | 0.005579 | 0.281576182 | Down |
| HSPA6 | Heat shock 70 kDa protein 6 | 0.0091283 | 0.281137584 | Down |
| RANBP2 | E3 SUMO-protein ligase RanBP2 | 0.0049998 | 0.280351998 | Down |
| FAM90A2P | Putative protein FAM90A2P | 0.000298 | 0.280000768 | Down |
| ALYREF | THO complex subunit 4 | 0.0016704 | 0.279428702 | Down |
| RBM26 | RNA-binding protein 26 | 0.0014855 | 0.277868339 | Down |
| TFPI2 | Tissue factor pathway inhibitor 2 | 0.0234251 | 0.277730169 | Down |
| POTEE | POTE ankyrin domain family member E | 0.0022835 | 0.276884815 | Down |
| SORCS1 | Isoform 2 of VPS10 domain-containing receptor SorCS1 | 0.0069557 | 0.275336942 | Down |
| **HIST1H2BB** | **Histone H2B type 1-B** | **0.0075408** | **0.274747455** | **Down** |
| ZNF599 | Zinc finger protein 599 | 0.0256513 | 0.273901856 | Down |
| MIA3 | Melanoma inhibitory activity protein 3 | 0.006317 | 0.272987354 | Down |
| DDX23 | Probable ATP-dependent RNA helicase DDX23 | 0.002912 | 0.272972711 | Down |
| NYAP2 | Neuronal tyrosine-phosphorylated phosphoinositide-3-kinase adapter 2 | 0.0033088 | 0.268945013 | Down |
| LUC7L3 | Luc7-like protein 3 | 0.0087484 | 0.266056779 | Down |
| SNX6 | Sorting nexin-6 | 0.0384501 | 0.265648405 | Down |
| DHX8 | ATP-dependent RNA helicase DHX8 | 0.0014855 | 0.26413426 | Down |
| RAB8A | Ras-related protein Rab-8A | 0.0184816 | 0.264092679 | Down |
| PKD1L2 | Polycystic kidney disease protein 1-like 2 | 0.0074218 | 0.263846374 | Down |
| TERF2IP | Telomeric repeat-binding factor 2-interacting protein 1 | 0.0151438 | 0.261950441 | Down |
| AP3B2 | AP-3 complex subunit beta-2 | 0.0026202 | 0.259646492 | Down |
| CCT7 | T-complex protein 1 subunit eta | 0.007541 | 0.257597694 | Down |
| KIF15 | Kinesin-like protein KIF15 | 0.0006576 | 0.257078424 | Down |
| RPS6KA3 | Ribosomal protein S6 kinase alpha-3 | 0.0190378 | 0.254801991 | Down |
| PTOV1 | Prostate tumor-overexpressed gene 1 protein | 0.0011138 | 0.25396953 | Down |
| DPYSL2 | Dihydropyrimidinase-related protein 2 | 0.0385108 | 0.25379213 | Down |
| PABPC1L2A | Polyadenylate-binding protein 1-like 2 | 0.0053846 | 0.250035378 | Down |
| PTBP2 | Polypyrimidine tract-binding protein 2 | 0.0118044 | 0.247902116 | Down |
| SRCIN1 | SRC kinase signaling inhibitor 1 | 0.0026275 | 0.247283711 | Down |
| CIRBP | Cold-inducible RNA-binding protein | 0.0071404 | 0.247245366 | Down |
| BMP1 | Bone morphogenetic protein 1 | 0.0291869 | 0.246773566 | Down |
| HNRNPK | Isoform 2 of Heterogeneous nuclear ribonucleoprotein K | 0.023176 | 0.246251775 | Down |
| BSN | Protein bassoon | 0.0004513 | 0.246048811 | Down |
| IK | Protein Red | 0.0053846 | 0.2453847 | Down |
| RPS16 | 40S ribosomal protein S16 | 0.0077812 | 0.245299547 | Down |
| GTPBP10 | GTP-binding protein 10 | 0.0047802 | 0.244590898 | Down |
| UCK2 | Uridine-cytidine kinase 2 | 0.0028415 | 0.241500644 | Down |
| GSC | Homeobox protein goosecoid | 0.027368 | 0.24125828 | Down |
| SDR42E1 | Short-chain dehydrogenase/reductase family 42E member 1 | 0.0298583 | 0.240179097 | Down |
| HIST1H1E | Histone H1.4 | 0.0005879 | 0.239510964 | Down |
| SMPD4 | Isoform 4 of Sphingomyelin phosphodiesterase 4 | 0.0325829 | 0.237977225 | Down |
| TBCE | Tubulin-specific chaperone E | 0.0030281 | 0.237596325 | Down |
| HIST1H1D | Histone H1.3 | 0.0005425 | 0.236939762 | Down |
| ZNF718 | Zinc finger protein 718 | 0.000174 | 0.234255629 | Down |
| NUMA1 | Nuclear mitotic apparatus protein 1 | 0.036715 | 0.232645989 | Down |
| CFH | Complement factor H | 0.0291869 | 0.232272239 | Down |
| SERBP1 | Isoform 3 of Plasminogen activator inhibitor 1 RNA-binding protein | 0.0004513 | 0.230642504 | Down |
| ZNF195 | Zinc finger protein 195 | 0.0117841 | 0.230520818 | Down |
| CASP5 | Caspase-5 | 0.002912 | 0.230150066 | Down |
| ASH1L | Histone-lysine N-methyltransferase ASH1L | 0.005579 | 0.229936498 | Down |
| TBC1D2B | TBC1 domain family member 2B | 0.0002465 | 0.227578783 | Down |
| BRWD1 | Bromodomain and WD repeat-containing protein 1 | 0.0019701 | 0.22674566 | Down |
| GBA3 | Cytosolic beta-glucosidase | 0.0049805 | 0.225306764 | Down |
| BOD1L1 | Biorientation of chromosomes in cell division protein 1-like 1 | 0.0139264 | 0.225257257 | Down |
| STK24 | Serine/threonine-protein kinase 24 | 0.0161933 | 0.223581643 | Down |
| **MYCBP** | **C-Myc-binding protein** | **0.016091** | **0.221368257** | **Down** |
| ZNF880 | Zinc finger protein 880 | 0.0117922 | 0.220615908 | Down |
| PLIN2 | Perilipin-2 | 0.0471261 | 0.219895003 | Down |
| CCT3 | T-complex protein 1 subunit gamma | 0.0003738 | 0.219107066 | Down |
| IKBIP | Inhibitor of nuclear factor kappa-B kinase-interacting protein | 0.0001546 | 0.21716434 | Down |
| HSPE1 | 10 kDa heat shock protein_ mitochondrial | 0.0004789 | 0.211822769 | Down |
| HSP90AB2P | Putative heat shock protein HSP 90-beta 2 | 0.0163136 | 0.210248795 | Down |
| GGN | Gametogenetin | 0.0047749 | 0.207695452 | Down |
| RPRD1A | Regulation of nuclear pre-mRNA domain-containing protein 1A | 0.002973 | 0.206424649 | Down |
| CBX4 | E3 SUMO-protein ligase CBX4 | 0.0399948 | 0.206350095 | Down |
| STARD9 | StAR-related lipid transfer protein 9 | 0.0003947 | 0.205113482 | Down |
| CCDC112 | Coiled-coil domain-containing protein 112 | 0.000174 | 0.203952535 | Down |
| SYT14L | Putative synaptotagmin-14-like protein | 0.0101048 | 0.199848642 | Down |
| IFFO1 | Intermediate filament family orphan 1 | 0.0001574 | 0.196894556 | Down |
| MARVELD3 | MARVEL domain-containing protein 3 | 0.0022835 | 0.189854561 | Down |
| CCDC167 | Coiled-coil domain-containing protein 167 | 0.0075918 | 0.189282039 | Down |
| RBM25 | RNA-binding protein 25 | 0.0003792 | 0.188876222 | Down |
| LRP5L | Low-density lipoprotein receptor-related protein 5-like protein | 0.003561 | 0.186226522 | Down |
| TRA2B | Transformer-2 protein homolog beta | 9.28E-05 | 0.18355611 | Down |
| PLXNA4 | Plexin-A4 | 0.0031573 | 0.181412751 | Down |
| HMCN2 | Hemicentin-2 | 0.0114744 | 0.181374913 | Down |
| PRKD1 | Serine/threonine-protein kinase D1 | 0.0014855 | 0.179795032 | Down |
| MIF | Macrophage migration inhibitory factor | 0.0060669 | 0.178422295 | Down |
| AKAP1 | A-kinase anchor protein 1_ mitochondrial | 0.000537 | 0.175806345 | Down |
| **YWHAB** | **14-3-3 protein beta/alpha** | **0.021164** | **0.175330625** | **Down** |
| TLL2 | Tolloid-like protein 2 | 0.0289527 | 0.174977668 | Down |
| ZNF160 | Zinc finger protein 160 | 0.0001574 | 0.174922298 | Down |
| PPAP2C | Lipid phosphate phosphohydrolase 2 | 0.0414068 | 0.170414368 | Down |
| ZNF248 | Zinc finger protein 248 | 0.0002465 | 0.169916219 | Down |
| HNRNPA1 | Heterogeneous nuclear ribonucleoprotein A1 | 0.0437737 | 0.169216615 | Down |
| EIF3A | Eukaryotic translation initiation factor 3 subunit A | 0.0003552 | 0.166071536 | Down |
| **ILF2** | **Interleukin enhancer-binding factor 2** | **0.0007045** | **0.165483307** | **Down** |
| CWF19L2 | CWF19-like protein 2 | 0.002973 | 0.165117272 | Down |
| TRAF3IP1 | TRAF3-interacting protein 1 | 0.0004122 | 0.164980413 | Down |
| ZNF701 | Zinc finger protein 701 | 0.0332363 | 0.16387371 | Down |
| SH3D21 | SH3 domain-containing protein 21 | 0.0123541 | 0.162851746 | Down |
| KIF6 | Kinesin-like protein KIF6 | 0.002912 | 0.162157528 | Down |
| ZNF326 | DBIRD complex subunit ZNF326 | 0.0031919 | 0.160186739 | Down |
| ZNF695 | Zinc finger protein 695 | 0.0023247 | 0.15452027 | Down |
| INTS4 | Integrator complex subunit 4 | 0.0036056 | 0.150613289 | Down |
| PFDN1 | Prefoldin subunit 1 | 0.0442948 | 0.150095414 | Down |
| COPS3 | COP9 signalosome complex subunit 3 | 0.0060564 | 0.14990941 | Down |
| CCDC80 | Coiled-coil domain-containing protein 80 | 0.0075918 | 0.148448939 | Down |
| KMT2A | Histone-lysine N-methyltransferase 2A | 9.28E-05 | 0.147846657 | Down |
| KIAA0895 | Isoform 3 of Uncharacterized protein KIAA0895 | 4.49E-05 | 0.145068234 | Down |
| PAPOLA | Poly(A) polymerase alpha | 0.0026275 | 0.143929111 | Down |
| C14orf166 | UPF0568 protein C14orf166 | 0.000522 | 0.143464971 | Down |
| ACTBL2 | Beta-actin-like protein 2 | 0.0395003 | 0.139057209 | Down |
| KDM3B | Lysine-specific demethylase 3B | 0.0005425 | 0.1379732 | Down |
| MAP7D3 | MAP7 domain-containing protein 3 | 9.28E-05 | 0.134438888 | Down |
| ACAT1 | Acetyl-CoA acetyltransferase_ mitochondrial | 0.0015063 | 0.133624529 | Down |
| GLUD1 | Glutamate dehydrogenase 1_ mitochondrial | 0.0004736 | 0.132197835 | Down |
| ANO9 | Anoctamin-9 | 0.0003051 | 0.1320651 | Down |
| CRISPLD2 | Cysteine-rich secretory protein LCCL domain-containing 2 | 0.0061932 | 0.131381536 | Down |
| POTEI | POTE ankyrin domain family member I | 0.0103074 | 0.128983911 | Down |
| C5orf51 | UPF0600 protein C5orf51 | 0.0081258 | 0.12830095 | Down |
| POLA2 | DNA polymerase alpha subunit B | 0.0021788 | 0.128199418 | Down |
| RPS10 | 40S ribosomal protein S10 | 0.0081968 | 0.125644244 | Down |
| SDHB | Succinate dehydrogenase [ubiquinone] iron-sulfur subunit_ mitochondrial | 0.0047802 | 0.124174456 | Down |
| TDRD10 | Tudor domain-containing protein 10 | 0.0003947 | 0.124168236 | Down |
| KDM1B | Lysine-specific histone demethylase 1B | 0.0006452 | 0.121622127 | Down |
| INTS6 | Integrator complex subunit 6 | 0.0008357 | 0.12050808 | Down |
| ZSCAN26 | Zinc finger and SCAN domain-containing protein 26 | 0.0054678 | 0.119696557 | Down |
| NUCKS1 | Nuclear ubiquitous casein and cyclin-dependent kinase substrate 1 | 0.0001574 | 0.118375603 | Down |
| NKX3-1 | Homeobox protein Nkx-3.1 | 0.0022835 | 0.118135837 | Down |
| HDGF | Hepatoma-derived growth factor | 0.0001546 | 0.116129521 | Down |
| DNAJB1 | DnaJ homolog subfamily B member 1 | 0.0117841 | 0.114670421 | Down |
| PRPF19 | Pre-mRNA-processing factor 19 | 0.0005813 | 0.11053606 | Down |
| CREBL2 | cAMP-responsive element-binding protein-like 2 | 0.0008357 | 0.10758772 | Down |
| NEFM | Neurofilament medium polypeptide | 0.0004904 | 0.105697364 | Down |
| UBE2M | NEDD8-conjugating enzyme Ubc12 | 0.0189077 | 0.104762931 | Down |
| MZB1 | Marginal zone B- and B1-cell-specific protein | 0.0216804 | 0.101425273 | Down |
| ZNF578 | Zinc finger protein 578 | 0.0169109 | 0.098644873 | Down |
| ZSCAN25 | Zinc finger and SCAN domain-containing protein 25 | 3.68E-05 | 0.095285685 | Down |
| BAG5 | BAG family molecular chaperone regulator 5 | 0.0225003 | 0.094454849 | Down |
| SART3 | Squamous cell carcinoma antigen recognized by T-cells 3 | 0.0101877 | 0.088202268 | Down |
| ARHGAP11A | Rho GTPase-activating protein 11A | 9.28E-05 | 0.085200828 | Down |
| CBX1 | Chromobox protein homolog 1 | 0.0062914 | 0.084942918 | Down |
| MDN1 | Midasin | 0.0001574 | 0.08406808 | Down |
| ALMS1P | Putative ALMS1-like protein | 0.0015855 | 0.080135977 | Down |
| NCOA5 | Nuclear receptor coactivator 5 | 0.0007328 | 0.075870104 | Down |
| IGIP | IgA-inducing protein homolog | 9.28E-05 | 0.075386627 | Down |
| CARD9 | Caspase recruitment domain-containing protein 9 | 0.0002939 | 0.06905999 | Down |
| **CCNB3** | **G2/mitotic-specific cyclin-B3** | **0.000174** | **0.069001562** | **Down** |
| RECQL5 | ATP-dependent DNA helicase Q5 | 0.0125921 | 0.066321386 | Down |
| ZNF616 | Zinc finger protein 616 | 0.0001574 | 0.064264593 | Down |
| NOL6 | Nucleolar protein 6 | 7.95E-05 | 0.061232818 | Down |
| WDR20 | WD repeat-containing protein 20 | 0.0022728 | 0.060850005 | Down |
| TPM3 | Isoform 2 of Tropomyosin alpha-3 chain | 3.75E-07 | 0.060175583 | Down |
| ZNF528 | Zinc finger protein 528 | 0.0048426 | 0.058061563 | Down |
| WDR59 | WD repeat-containing protein 59 | 9.28E-05 | 0.057323159 | Down |
| DDX17 | Probable ATP-dependent RNA helicase DDX17 | 0.0004966 | 0.055470272 | Down |
| TRMU | Mitochondrial tRNA-specific 2-thiouridylase 1 | 0.0002736 | 0.054910693 | Down |
| 40422 | Septin-10 | 0.0008024 | 0.049054726 | Down |
| SPTY2D1 | Protein SPT2 homolog | 0.0005838 | 0.046358961 | Down |
| CHD7 | Chromodomain-helicase-DNA-binding protein 7 | 0.0007874 | 0.045414526 | Down |
| GALNT10 | Polypeptide N-acetylgalactosaminyltransferase 10 | 1.17E-05 | 0.044029018 | Down |
| ZMYM3 | Zinc finger MYM-type protein 3 | 0.011226 | 0.039379452 | Down |
| C6orf222 | Uncharacterized protein C6orf222 | 0.0031919 | 0.024515883 | Down |
| AACS | Acetoacetyl-CoA synthetase | 0.0003552 | 0.016551628 | Down |

Additional protocols:

**Preparation of samples for mass spectrometry.** Cell pellet was resuspended in 600 μl of 1% ammonium deoxycholate (ADC) (Sigma-Aldrich) and placed in a Homogenizer (Beadblaster 24 microtube homogenizer). After homogenized, the samples were centrifugated at 16000 g for 20 min and the supernatants were transferred into new labelled Eppendorf tubes. The samples lysate was either used for protein concentration experiment or stored at -80°C until required. To determine the concentration of protein, a bicinchoninic acid assay was used. BSA was used as a standard protein for quantifying the amount of protein in the samples. The BCA working solution was prepared by mixing 1.35 ml of solution A (0.8 g of sodium carbonate monohydrate and 0.16 mg of tartaric acid into 10 ml of water, pH=11.25 with NaOH), 1.25 ml of B solution (1 g of bicinchoninic acid in 25 ml of water), and 50 μl of C solution (40 mg of copper sulphate in 1 ml of water). The top wells (A1 and A2) were loaded with 120 μl of water and 80 μl of BSA as a standard. Then, 100 μl of water were added to the wells from B1 to H1 and B2 to H2. A serial dilution was made by transferred 100 μl from the top wells (standard) except the bottom wells (H1 and H2) were left the same. To load the samples, each well was loaded with 98 μl of water and 2 μl of sample in triplicates followed by adding 100 μl of the working solution. After the plate was incubated for an hour at 65°C (Thermo Multiskan Ascent), the results were read at 562 nm by a spectrophotometer. Following the estimation of protein concentration using the BCA assay, equivalent amounts of protein (1000μg) were transferred to Microcon Centrifugal Filter units (Milipore) followed by adding Ammonium bicarbonate (ABC) to dilute 1000 μg of protein into a final volume of 1000 μl. Then, the samples were incubated for 1 hour at 65°C after adding Dithiothreitol (Sigma-Aldrich) (15.4 mg of dithiothreitol into 100 μl of 50 mmol ammonium bicarbonate) at final concentration of 20 mM/l. After that, Iodoacetamide (Sigma-Aldrich) (18.5 mg of iodoacetamide into 200 μl of 50 mmol ammonium bicarbonate) at a concentration of 40 mM/l followed by incubation for 1 hour in the dark at room temperature. Next, 1 μg/μl Trypsin (20 μl of 50 mmol ammonium bicarbonate into a vial of 20 μg of trypsin (Roche)) was added to samples at a 1:25 ratio to protein content, 1 μg/μl and left overnight at 37°C. Next day, the activity of trypsin was stopped by adding 0.1% of formic acid (FA) to the samples. Empore™ Solid Phase Extraction Cartridges (3M) were used for peptide extraction. After labelled and washed with ethanol, the columns were emptied by gravity. Then, the columns were washed four times with 0.1% FA. After that, the samples were placed and flowed through the columns by gravity followed by washing via 0.1% FA four times. To collect the samples, new labelled Eppendorf were prepared and 600 μl of 60 % Acetonitrile (Sigma-Aldrich) was applied to the columns followed by adding 600 μl of 80% acetonitrile (Sigma-Aldrich) for elution by gravity. Following that, samples were centrifugated for 90 minutes by speed vacuum centrifuge (Thermo, RC1010). Following snap freezing in liquid nitrogen, samples were placed in a freeze dryer (LyoDry Compact Benchtop, MechaTech) and left overnight. The following day, samples were reconstituted with 30 μl of 0.1% FA and stored at -80°C until required. After that, an o-Phthaladehyde (OPA) assay was applied to identify the concentration of peptide in the samples. Firstly, in the standard wells A1 and A2 of the plate 90 μl of water and 10 μl of 1 mg/ml ROCK peptide (20 mg of OPA (Fisher Scientific) were added to 250 μl of Dimethylformamide (Sigma-Aldrich)). Secondly, 50 μl of water was added into wells B1 to H1 and B2 to H2. Next, the standard wells were serially diluted in 50 μl amounts, excluding the last wells, H1 and H2. In the sample wells, 49 μl of water and 1 μl of each sample were then added in triplicates. Then, to all of the wells, 100 μl of a boric acid mixture including 200 μl of ROCK peptide, 20 ml of boric acid, and 40 μl of mercaptoethanol was added, followed by a 5-minute incubation at room temperature before reading at an excitation of 340 nm and emission of 490 nm. Lastly, 5 μl of each sample were transferred to labelled 32 mm glass screw neck vials and diluted to be 1 μg /μl using 0.1% FA and alcohol dehydrogenase.
